# Achieving Dynamic Circularly Polarized Luminescence with 2D Hydrogen‐Bonded Organic Frameworks

**DOI:** 10.1002/advs.202500789

**Published:** 2025-03-07

**Authors:** Li Meng, Dian‐Xue Ma, Zhong‐Qiu Li, Tongling Liang, Zili Chen, Yu‐Wu Zhong

**Affiliations:** ^1^ Beijing National Laboratory for Molecular Sciences CAS Key Laboratory of Photochemistry CAS Research/Education Center for Excellence in Molecular Sciences Institute of Chemistry Chinese Academy of Science 2 Bei Yi Jie, Zhong Guan Cun, Haidian District Beijing 100190 China; ^2^ School of Chemistry and Life Resources Renmin University of China 59# Zhongguancun Street, Haidian District Beijing 100872 China; ^3^ Institute of Molecular Engineering Plus College of Chemistry Fuzhou University Fuzhou 350108 China

**Keywords:** chirality transfer, circularly polarized luminescence, host‐guest system, hydrogen‐bonded organic frameworks, organic crystals

## Abstract

Significant advances have been made in the preparation and application of luminescent hydrogen‐bonded organic frameworks (HOFs) in recent years. These materials exhibit unique structural flexibility in accommodating guest molecules, rendering them promising candidates for dynamic applications. Herein, a 2D HOF material is presented, constructed by the binary assembly of 5,5′‐bis(azanediyl)oxalyl diisophthalic acid and tetra(pyrid‐4‐ylphenyl)ethylene to achieve dynamic circularly polarized luminescence (CPL) with the aid of chiral guests. Efficient chirality transfer and CPLs are realized by incorporating chiral carvone molecules into the 1D channels of HOFs through hydrogen bonding. As a result of the interlayer contraction upon guest desorption, these HOF materials display dynamic CPLs with emission colors varying from cyan to yellow. The method of the hydrogen bonding‐enhanced host‐guest chirality transfer in combination with the guest desorption‐triggered interlayer contraction provides a simple method to achieve dynamic chiroptical properties with porous materials.

## Graphical abstract

Efficient chirality transfer and circularly polarized luminescence (CPL) are realized by incorporating chiral carvone molecules into the 1D channels of luminescent hydrogen‐bonded organic frameworks (HOFs). These materials display dynamic CPLs with varied emission colors via a guest desorption‐triggered interlayer contraction mechanism. Interestingly, only the chiral molecules directly binding to the HOF skeletons through hydrogen bonding influence the CPL magnitude.

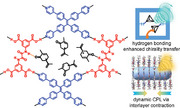

## 1 Introduction

Highly crystalline materials with well‐defined porous structures, including metal‐organic frameworks (MOFs),^[^ ^1^ ^]^ covalent organic frameworks (COFs),^[^ ^2^ ^]^ and hydrogen‐bonded organic frameworks (HOFs),^[^ ^3^ ^]^ have received intensive and extensive attention in recent decades. Among them, HOFs are constructed mainly based on the noncovalent hydrogen‐bonding interactions and possess appealing features such as easy preparation and excellent self‐healing properties, making them potentially useful for gas storage and separation,^[^ ^4^ ^]^ proton conduction,^[^ ^5^ ^]^ enzyme encapsulation,^[^ ^6^ ^]^ and so on.^[^ ^7^ ^]^ For instance, HOFs are used to anchor non‐noble metal ions to achieve efficient photocatalytic hydrogen production.^[^ ^8^ ^]^ The reversible capture and release of iodine (I_2_) within the cavities of HOFs allows the fabrication of Li‐I_2_ and Na‐I_2_ batteries.^[^ ^9^ ^]^ Room‐temperature phosphorescence materials have been prepared by incorporating phosphors into the porosities of HOFs.^[^ ^10^ ^]^ In addition, HOFs are demonstrated to show switchable optical and luminescent properties in response to different liquid environments.^[^ ^11^ ^]^ These properties can undergo significant changes via the exchange of the guest molecules within HOF channels, leading to color‐tunable luminescences.^[^ ^12^ ^]^

**Figure 1:**
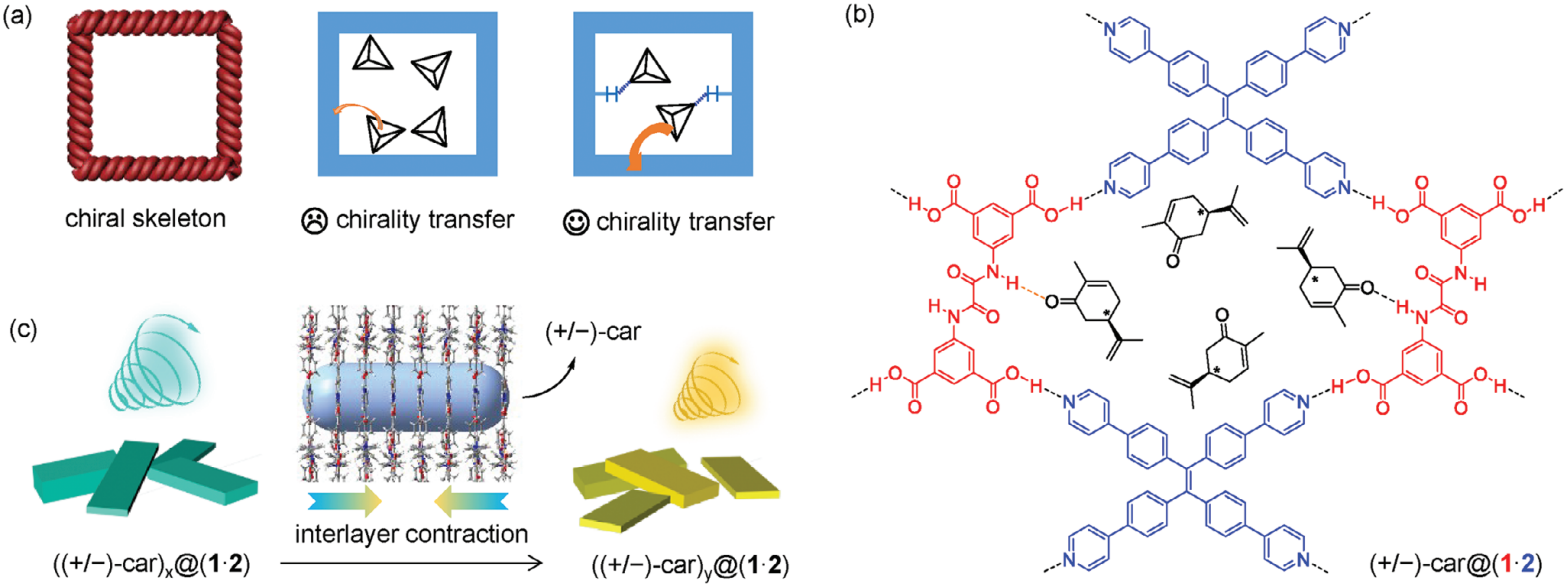
The design concept of this work. a) Three types of chiral frameworks to realize CPL. b) Host‐guest chiral HOF reported in this work. c) Schematic diagram showing dynamic CPLs via interlayer contraction. car = carvone.

Among luminescent materials, those showing circularly polarized luminescence (CPL) hold great promise for applications in low‐power displays and encrypted information storage.^[^ ^13^ ^]^ To date, a large number of CPL‐active materials have been developed.^[^ ^14^ ^]^ One challenging task in this field is the realization of tunable CPLs with both high emission quantum yield (*Φ*) and luminescence dissymmetry factor (*g*_lum_).^[^ ^15^ ^]^ Besides the conventional helical assemblies of organic and inorganic materials, the incorporation of chiral moieties into porous luminescent crystalline frameworks provides a simple means to achieve this goal.^[^ ^16^ ^]^ In general, there are two methods to endow framework materials with CPL activity (**Figure** **1**a). Luminescent frameworks constructed with chiral skeletons represent a classical method. A considerable amount of chiral MOFs and COFs have been prepared using this method to show efficient CPLs, taking advantage of the efficient chirality transfer and amplification among rigid subcomponents.^[^ ^17^ ^]^ This method however heavily relies on the availability of the chiral building blocks. The incorporation of chiral guest molecules into the cavity of luminescent frameworks provides another modular method to prepare CPL‐active materials, which is conceptually simple yet more difficult to realize. Due to the lack of strong and directional host‐guest interaction, chiral guest molecules tend to be arranged in a disordered manner with a certain degree of fluidity in the cavity, leading to insufficient chirality transfer and thus poor CPL performance. To date, only very limited examples have been successfully implemented with this chiral‐guest method.^[^ ^18^ ^]^

We envisage that, if chiral guest molecules could be bound to the luminescent skeleton through directional and relatively strong hydrogen bonding interaction, framework materials with efficient chirality transfer and excellent CPL performance may be obtainable. In order to prove this concept, we have selected 5,5′‐bis(azanediyl)oxalyl diisophthalic acid (**1**) and tetra(pyrid‐4‐ylphenyl)ethylene (**2**) to construct the achiral luminescent binary 2D HOF material (**1**⋅**2**) (Figure 1b). Compound **1** is a known building block for the construction of binary HOFs with another complementary pyridine‐terminated ligand.^[^ ^19^ ^]^ The bridging amide groups of **1** are believed to provide suitable functional sites to form hydrogen bonds with chiral guest molecules for our purpose.^[^ ^20^ ^]^ Compound **2** is an archetypal pyridine‐terminated fluorophore with aggregation‐induced emission (AIE) behavior, which is expected to bring remarkable solid‐state luminescence properties to the porous framework.^[^ ^21^ ^]^ After screening a number of chiral guest molecules, we found that the incorporation of chiral (+/−)‐carvone (car) molecules within the 1D channels of the (**1**⋅**2**) HOF successfully gave the (+/−)‐car@(**1**⋅**2**) HOF composite with distinct CPL activity. Interestingly, depending on the amount of guest loading, these HOFs display dynamic CPLs with varied emission colors as a result of interlayer contraction (Figure 1c). This work presents a new and simple concept to prepare framework materials with dynamic CPLs and it is believed to greatly stimulate the development of HOFs with CPL activity.^[^ ^22^ ^]^

## 2 Results and Discussion

**Figure 2:**
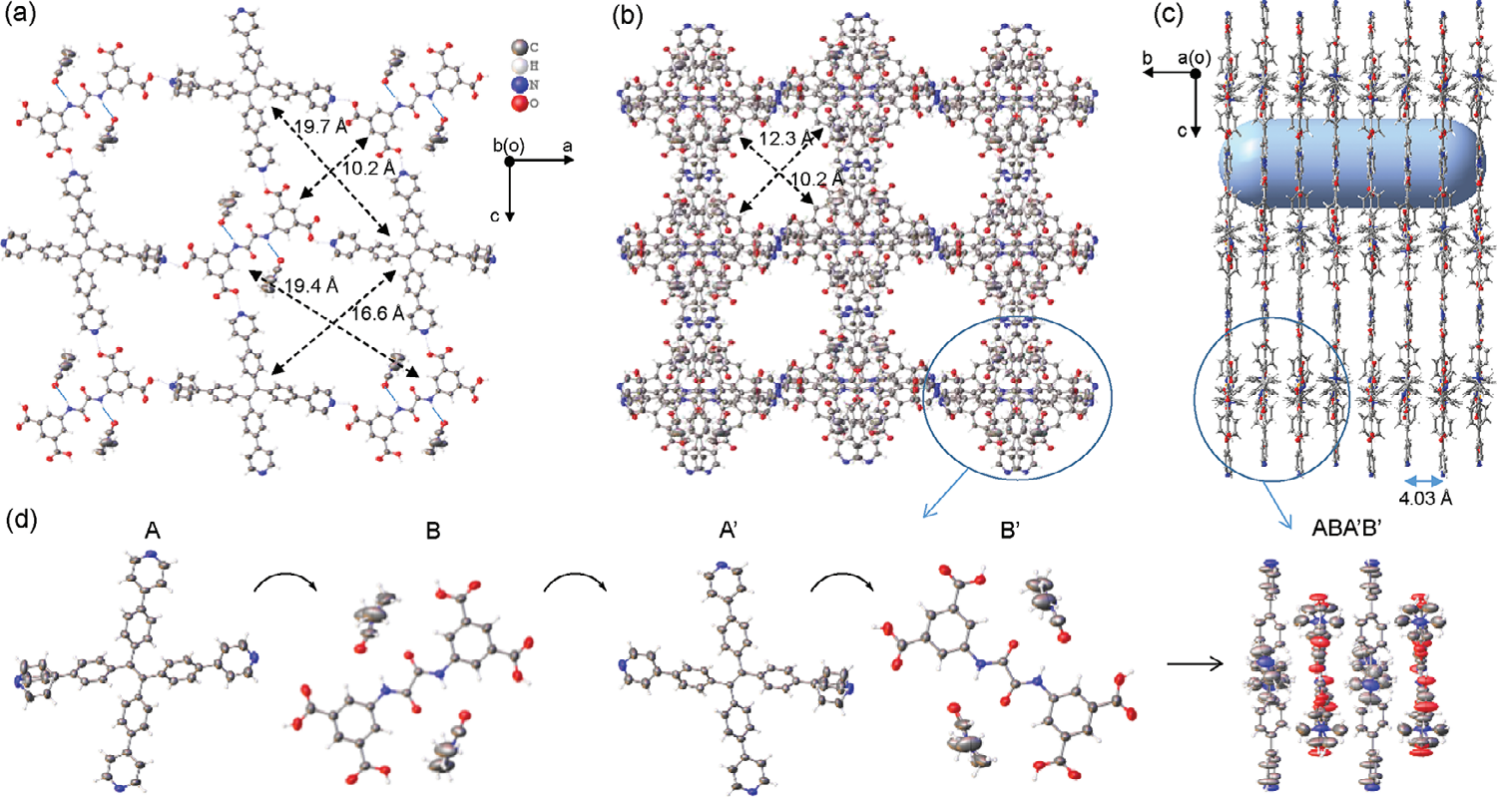
Single crystal X‐ray analysis of DMF@(**1**·**2**). a) Thermal ellipsoid plot at 30% probability of one layer structure. b,c) Multilayer structure viewed from the *b* and *a* axis, respectively. d) A repeating unit of ABA'B’ four‐layer stacking.

The reaction of **1** and **2** in dimethylformamide (DMF) at 90 °C resulted in the formation of green crystals which were later characterized as the binary 2D HOF cocrystal of **1** and **2** with included DMF molecules (Figure S1, Supporting Information). This material is abbreviated as DMF@(**1**·**2**). Single crystal X‐ray analysis shows that the 1D channels of this crystal are filled with large amounts of solvent molecules. Most of these disordered solvent molecules are squeezed by the PLATON program and only those directly bound to the framework skeleton by hydrogen bonds are retained. The resulting structure has a composition of (DMF)_2_@(**1**·**2**), which crystallizes in the orthorhombic space group *P*bcn and exhibits a perfect 2D framework arrangement (**Figure** **2**; Table S1, Supporting Information). The crystal packing shows a well‐defined lamellar structure when viewed along the crystallographic *a* axis, with an interlayer distance of ≈4.03 Å (Figure 2c). Within each layer, each molecule of **1** (or **2**) is linked to four molecules of **2** (or **1**) via carboxylic acid‐pyridine hydrogen bond interactions with a H···N distance of 1.725–1.864 Å (Figure 2a). This arrangement prompts the equivalent combination of **1** and **2** to form a continuous 2D porous framework with two types of pores with a dimension of 10.2 Å × 19.7 Å and 16.6 Å × 19.4 Å, respectively. Within the latter larger pore, two DMF molecules are linked to the amide group of **1** via the C = O···H‐N hydrogen bond interactions with an O···H distance of 2.236 Å. These monolayers are stacked in an ABA'B’ fashion to yield the HOF structure with 1D channels with a dimension of 10.2 Å × 12.3 Å (Figure 2b,d).

**Figure 3:**
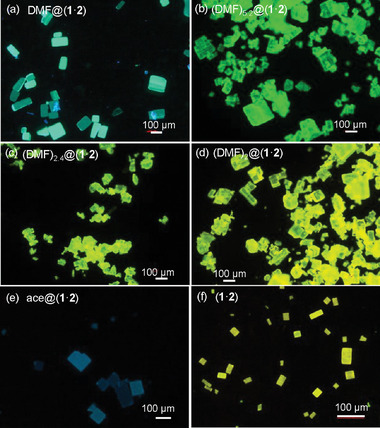
Fluorescence microscopy images of different microcrystals. ace = acetone. Crystal ace@(**1**·**2**) was obtained by solvent exchange of DMF@(**1**·**2**) with acetone. Solvent‐free crystal (**1**·**2**) was obtained from ace@(1·2) after solvent desorption.

The crystals of DMF@(**1**·**2**) exhibit a cuboid morphology with lengths of 50–100 µm and thickness of 10–50 µm (Figure S2, Supporting Information). The original crystals of DMF@(**1**·**2**) show bright cyan emissions. Interestingly, after gradual solvent desorption, the emission colors of these crystals change to green and yellow successively (**Figure** **3**; Figure S3, Supporting Information). The contents of the DMF solvents contained in the crystals are quantified by ^1^H NMR spectral analysis (Figure S4, Supporting Information). For instance, the crystals of (DMF)_5.2_@(**1**·**2**) display bright green fluorescence at 504 nm and those of (DMF)_2_@(**1**·**2**) are yellow emissive (Figure 3; Figures S3 and S5, Supporting Information), which is distinctly red‐shifted compared to the blue emission of chromophore **2** (Figure S6, Supporting Information).^[^ ^21c^ ^]^ This suggests that the luminescence in these binary HOF crystals may possess a character of charge transfer between **2** and **1**.

Because of the relatively high boiling point of DMF, it is somewhat difficult to completely remove the DMF solvents from the crystals of DMF@(**1**·**2**). Alternatively, DMF@(**1**·**2**) is transformed into ace@(**1**·**2**) (ace = acetone) via solvent exchange by immersing DMF@(**1**·**2**) into the low‐boiling‐point solvent acetone. The crystals of ace@(**1**·**2**) possess similar crystal shape and lattice parameters as those of DMF@(**1**·**2**) (Table S1, Supporting Information). Single‐crystal X‐ray analysis of ace@(**1**·**2**) reveals the presence of hydrogen bond interactions between the acetone C = O group and the N‐H group of **1** with an O···H distance of 2.45 Å (Figure S7, Supporting Information). When immersed in acetone, the crystals of ace@(**1**·**2**) show relatively weak blue emissions (Figure 3e). The emission colors of these crystals quickly change to yellow in the air at rt as a result of the desorption of acetone (Video S1, Supporting Information). After degassing under vacuum at 60 °C for a few hours, the solvent‐free crystals (**1**·**2**) are obtained, which show bright yellow luminescence (Figure 3f). NMR and elemental analyses confirm that this material retains an equimolar combination of **1** and **2** (Figure S8, Supporting Information).

**Figure 4:**
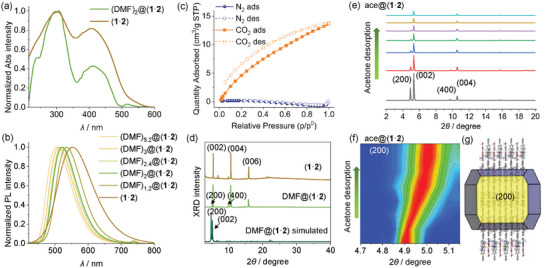
Physical characterizations of HOF microcrystals. a) Absorption spectra of (DMF)_2_@(**1**·**2**) and (**1**·**2**). b) Photoluminescence (PL) spectra of (DMF)_x_@(**1**·**2**) and (**1**·**2**). λ_ex_ = 365 nm. c) N_2_ (77 K) and CO_2_ (273 K) adsorption (ads) and desorption (des) profile of (**1**·**2**) microcrystals. d) PXRD patterns of (**1**·**2**), DMF@(**1**·**2**), and the simulated pattern of DMF@(**1**·**2**). e) Changes of PXRD patterns and f) evolution of the normalized intensity of the (200) peak of ace@(**1**·**2**) upon the desorption of acetone. g) Simulated growth morphology of (**1**·**2**).

**Figure** **4**a shows a comparison of the absorption spectra of the DMF‐incorporated crystal DMF@(**1**·**2**) and the solvent‐free crystal (**1**·**2**). A distinct red‐shift of the absorption spectrum is observed for (**1**·**2**) with respect to DMF@(**1**·**2**). This change of the absorption spectra is consistent with the gradual emission spectral red‐shift of DMF@(**1**·**2**) upon solvent desorption (Figure 4b; Figure S5, Supporting Information), which is caused by the desorption‐triggered interlayer contraction of the HOF crystal as discussed below. The crystals of (DMF)_2_@(**1**·**2**) have a moderate fluorescence quantum yield (*Φ*_FL_) of 28.4%. In contrast, the solvent‐free crystals of (**1**·**2**) exhibit a high *Φ*_FL_ of 92.5% with an emission maximum wavelength (*λ*_emi,max_) of 543 nm. These crystals display an emission decay lifetime (*τ*) in the range of 2–5 ns (Table S2, Supporting Information).

Powder X‐ray diffraction (PXRD) analyses show that the DMF‐included crystal and the solvent‐free crystal possess similar diffraction patterns, with distinct peaks corresponding to the (002), (004), and (006) planes being observed (Figure 4d). However, the diffraction intensities of the solvent‐free crystal (**1**·**2**) are relatively weaker with respect to those of DMF@(**1**·**2**), suggesting that solvent desorption leads to the deterioration of crystallinity to some degree.

The in‐situ PXRD spectral change analysis is further performed on the ace@(**1**·**2**) crystals upon the desorption of acetone. In addition to (002) and (004) peaks, the ace@(**1**·**2**) crystals display sharp and distinct (200) and (400) diffractions (Figure 4e). Upon the desorption of acetone, the intensities of these diffractions gradually decrease. Besides, a distinct shift toward the higher angle region (2*θ* changes from 4.91 to 5.02°) and signal broadening is observed for the (200) peak (Figure 4f). The (002) diffraction displays a minor shift toward the higher angle region, with the 2*θ* value changing from 5.30 to 5.33° (Figure S9, Supporting Information). According to the simulated growth morphology of the crystal, the (002) and (200) planes are the principal exposed crystal planes of ace@(**1**·**2**), and they depict the lamellar stacking of 2D HOF layers (Figure 4g; Figure S9, Supporting Information). The higher‐angle shift of these diffractions points to the decrease in the interlayer distance among 2D HOFs upon the guest desorption. Such a desorption‐triggered interlayer contraction leads to a more compact interlayer molecular packing and thus the redshifts of the emission spectrum of (**1**·**2**) with respect to those containing a high loading of guest molecules such as acetone, DMF, and other guests discussed below.

The active HOF of (**1**·**2**) shows a high gas adsorption selectivity of CO_2_ over N_2_. The N_2_ gas is barely adsorbed at 77 K, suggesting that the nonpolar N_2_ molecules could rarely be adsorbed by the polar channels.^[^ ^23^ ^]^ In contrast, it displays a type‐I CO_2_ adsorption isotherm at 273 K (Figure 4c), in agreement with its microporous structure. The Brunauer‐Emmett‐Teller (BET) surface area is determined to be 151.9 m^2^ g^−1^ based on the CO_2_ adsorption isotherm. Thermogravimetric analysis (TGA) shows that these framework structures possess good thermal stability. The crystals of (DMF)_2_@(**1**·**2**)·crystals exhibit ≈7% weight loss between 120 and 210 °C due to the desorption of DMF molecules. The framework structures begin to decompose when the temperature exceeds ≈350 °C (Figure S10, Supporting Information).

The above findings illustrate that the fluorescent color of the (**1**·**2**) HOF crystals can be readily modulated by guest exchange and desorption, while their ordered porous structures are well preserved. We therefore infer that the in situ incorporation of chiral molecules within HOFs has the potential to induce CPL characteristics by chirality transfer. Specifically, (+/−)‐car is proved to be a suitable chiral inducer to achieve this goal. The carvone molecule contains a carbonyl group, allowing it to interact strongly with the HOF structure by hydrogen bonding interaction. In addition, the (**1**·**2**) HOF crystals are essentially insoluble in carvone as a solvent, allowing the easy preparation of the desired host‐guest crystals by a simple soaking method.

When the ace@(**1**·**2**) host‐guest crystals are soaked in (+)‐ or (−)‐carvone for 96 h at 50 °C, the emission color gradually changes from blue to cyan. The successful exchange of the acetone guests by (+/−)‐carvone is confirmed by a number of evidence shown below. The obtained microcrystals, abbreviated as (+/−)‐car@(**1**·**2**) crystals, exhibit cyan emissions with *λ*_emi,max_ of 495 nm and appear as rectangular microcubes with similar shape and sizes as ace@(**1**·**2**) (Figure S11, Supporting Information). A single crystal of (+)‐car@(**1**·**2**) has been obtained, though the crystal quality is somewhat low due to the presence of twin structures and structural disorder caused by the guest molecules, and the poor diffraction at high angles (Figure S12, Supporting Information). Nevertheless, after squeezing out some highly‐disordered solvent molecules, a crystal structure with a composition of (**1**·**2**)_4_·((+)‐car)_8_ and a triclinic chiral space group *P*1 is obtained (Table S1 and Figure S13, Supporting Information). It is confirmed that the basic (**1**·**2**) HOF structure is retained and the (+)‐carvone molecules are encapsulated within the 1D channel cavities of the crystal. The presence of C = O···H‐N hydrogen bond interactions with an O···H distance of 2.26 Å is observed between carvone and the amide groups of **1**.

The freshly prepared (+)‐car@(**1**·**2**) crystal has a composition of ((+)‐car)_5.5_@(**1**·**2**) as determined by ^1^H NMR analysis (Figure S14, Supporting Information). At ambient conditions, the desorption of carvone guests is quite slow. After storing for 5 days at rt, the composition of the crystal becomes ((+)‐car)_3.2_@(**1**·**2**) (Figure S15, Supporting Information). A crystal with a composition of ((+)‐car)_1.9_@(**1**·**2**) is obtained after around a month. The desorption rate of carvone is speeded up at high temperatures. TGA analysis shows that the (+)‐car@(**1**·**2**) crystals begin to lose carvone molecules at ≈70–80 °C (Figure S16, Supporting Information). After heating at 80 °C for 24 h, the composition of the crystal becomes ((+)‐car)_2.5_@(**1**·**2**) (Figure S17, Supporting Information), allowing the preparations of the samples of ((+)‐car)_x_@(**1**·**2**) containing different amounts of (+)‐carvone guests within tens of hours. ^1^H NMR analysis shows that no proton signal of (+)‐carvone is present in the sample after heating at 80 °C under vacuum for 1 month (Figure S17c, Supporting Information). This sample is abbreviated as ((+)‐car)_<0.01_@(**1**·**2**) considering that the residual content of (+)‐carvone within the HOF structure, if any, is negligible. The HOF crystals of ((−)‐car)_x_@(**1**·**2**) containing different amounts of (−)‐carvone guests are prepared in a similar fashion. PXRD analysis confirms that the basic HOF structure and crystallinity are maintained in the crystals of ((+/−)‐car)_x<0.01_@(**1**·**2**) (Figure S18, Supporting Information).

**Figure 5:**
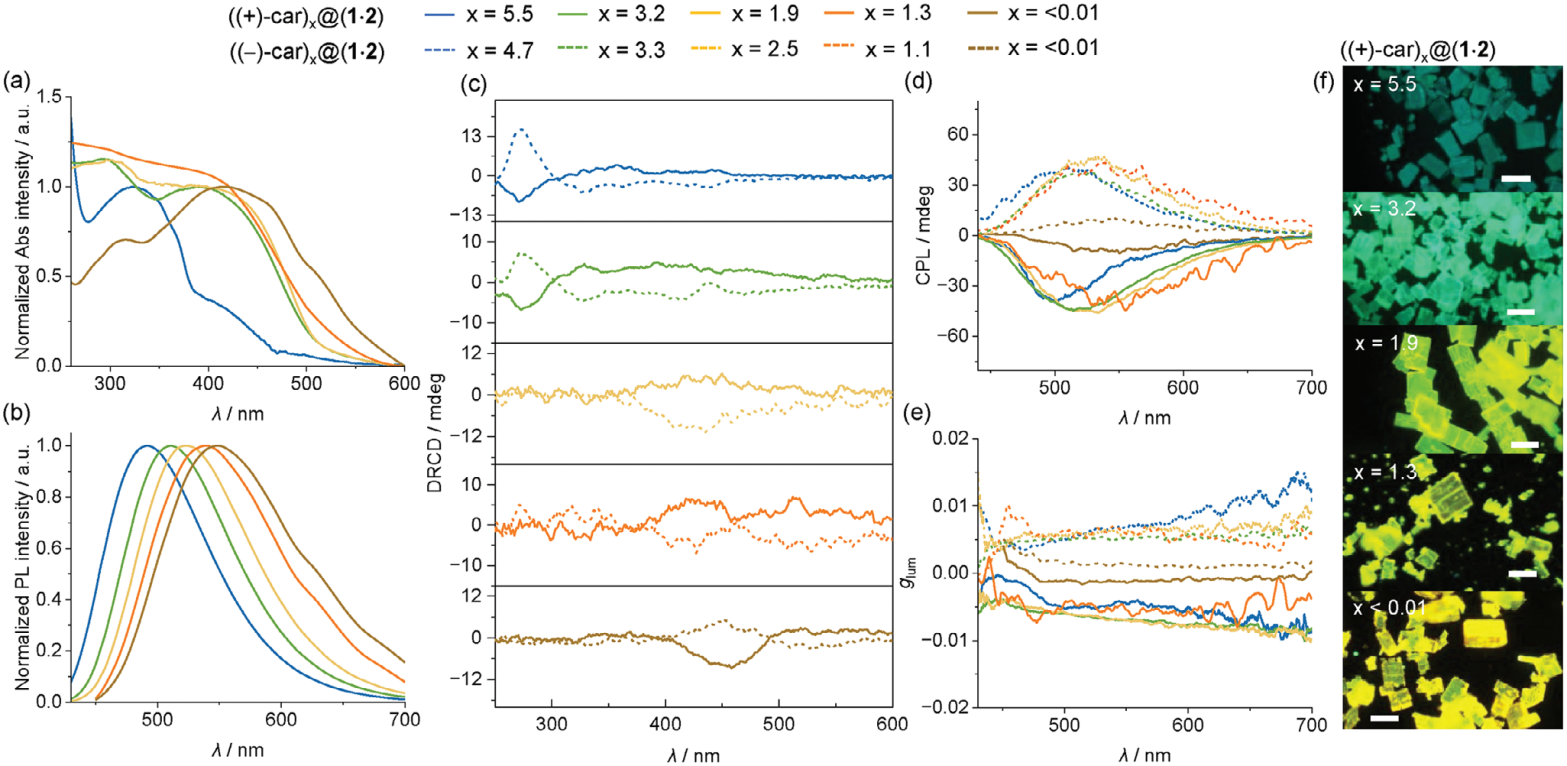
Optical and chiroptical properties of microcrystals. a) Absorption and b) emission spectra of ((+)‐car)_x_@(**1**·**2**) microcrystals. c) DRCD spectra, d) CPL spectra, and e) plot of *g*_lum_ versus emission wavelength of ((+)‐car)_x_@(**1**·**2**) and ((−)‐car)_x_@(**1**·**2**) microcrystals. f) Fluorescence microscopy images of ((+)‐car)_x_@(**1**·**2**) microcrystals. Scale bar: 100 µm. The excitation wavelength is 365 nm.

The content of the carvone guest has a significant impact on the optical properties of these host‐guest HOF materials. When carvone is gradually desorbed, the absorption and emission spectra of the obtained microcrystals are distinctly red‐shifted (**Figure** **5**a,b). In particular, the microcrystals of ((+)‐car)_x_@(**1**·**2**) with x of 5.5, 3.2, 1.9, 1.3, and <0.01 show *λ*_emi,max_ of 495, 510, 533, 540, and 545 nm, respectively. This is in agreement with the emission color change of these microcrystals from cyan to yellowish green upon guest desorption (Figure 5f). The *λ*_emi,max_ of ((+)‐car)_<0.01_@(**1**·**2**) is very similar to the guest‐free sample of (**1**·**2**) obtained from the solvent desorption of ace@(**1**·**2**) (Table S2, Supporting Information).

The guest‐free crystals of (**1**·**2**) show no diffused reflection circular dichroism (DRCD) and CPL signals at all (Figure S19, Supporting Information). Gratifyingly, the crystals of ((+/−)‐car)_x_@(**1**·**2**) with comparable amounts of (+)‐ or (−)‐carvone display roughly mirror‐imaged DRCD signals (Figure 5c). The DRCD signals in the short wavelength region (< 300 nm) are attributed to the chiral carvone guests. The appearance of the DRCD signals in the wavelength region between 300–600 nm reflects an efficient chirality transfer from the chiral carvone guests to the HOF framework. With decreasing amounts of the chiral guest molecules, the DRCD signals are distinctly red‐shifted. This is consistent with the trend of the absorption spectral changes (Figure 5a). In comparison, the solutions of (+/−)‐carvone or those of the host‐guest chiral crystals dissolved in EtOH show no circular dichroism (CD) signals in this long wavelength region (Figure S20, Supporting Information).

The crystals of ((+/−)‐car)_x_@(**1**·**2**) with different comparable amounts of (+)‐ or (−)‐carvone display mirror‐imaged CPL signals (Figure 5d). Negative CPL signals are observed with (+)‐carvone, while those with (−)‐carvone exhibit positive CPL signals. The crystals of ((+/−)‐car)_x_@(**1**·**2**) with x of 1.1–5.5 show similar CPL intensities despite their significantly different emission colors with different x values. These crystals have a luminescence asymmetry factor |*g*_lum_| of ≈5.0 × 10^−3^ at the CPL maximum (Figure 5e; Table S2, Supporting Information). When the content of carvone (the x value) is further reduced to 0.3–0.5, the obtained crystals exhibit similar yellowish green emission to that of (**1**·**2**), with a |*g*_lum_| of ≈4.0 × 10^−^^3^ (Figure S21 and Table S2, Supporting Information). In contrast, the CPL intensities of ((+)‐car)_<0.01_@(**1**·**2**) crystals are much weaker and they have a |*g*_lum_| of ≈1.5 × 10^−3^. Considering that each pair of (**1**·**2**) can accommodate two carvone molecules by hydrogen bonding, it is reasonable to deduce that only the chiral carvone molecules directly bound to the HOF framework make major contributions to the excited‐state chirality transfer and the CPL intensities of these host‐guest materials. The remaining carvone guests without direct hydrogen bonding with the HOF structure mainly influence the CPL wavelengths by a desorption‐triggered interlayer contraction mechanism as described above. In addition, the observation of the mirror‐imaged DRCD and CPL signals in ((+)‐car)_<0.01_@(**1**·**2**) crystals is suggestive of a chirality memory and/or amplification effect in this system. Furthermore, the crystals of ((+/−)‐car)_3.0_@(**1**·**2**) and ((+)‐car)_<0.01_@(**1**·**2**) retained their DRCD and CPL signals after one year of storing in a sealed container at room temperature (Figure S22, Supporting Information). This is consistent with the ^1^H NMR analysis, showing that the desorption of carvone is negligible when these crystals are stored in an airtight environment (Figure S23, Supporting Information). These results further highlight the stability and chirality memory of this host‐guest system.

Regarding the opposite chirality observed between the DRCD and CPL spectra, we conducted additional measurements of CD in the transmission mode (Figures S24 and S25, Supporting Information). These measurements show that the CD chirality at long wavelengths in the transmission mode is indeed consistent with that of CPL. The discrepancy between DRCD and CD spectra is likely caused by the differences between transmission and diffuse reflectance measurements, which have also been observed in the literature.^[^ ^24^ ^]^ Control experiments suggest that the above DRCD, CD, and CPL signals of these microcrystals are reliable and the contribution from linear anisotropy and light scattering can be neglected (Figures S26–S31, Supporting Information). In addition, when (+)‐ or (−)‐cinene (cin), which has a similar structure as carvone but lacks the carbonyl group, is used as the chiral solvent to prepare potential (+/−)‐cin@(**1**·**2**) crystals from ace@(**1**·**2**), the obtained materials are essentially DRCD‐ and CPL‐inactive (Figures S32–S34, Supporting Information). This suggests that the presence of the hydrogen bonding in (+/−)‐car@(**1**·**2**) crystals is critical in achieving the efficient CPL.

**Figure 6:**
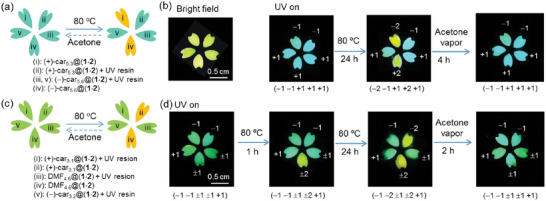
Optical applications of microcrystals. a,c) Schematic representation of a reversible process by heating and acetone‐fumigating a flower pattern with five petals (i–v) consisting of different compositions of microcrystals indicated. b,d) Images taken during the implementation of the process are shown in panels (a) and (c) respectively. Each image represents a five‐number code shown in the parentheses, in which each number is sequentially determined by the optical state of the petal (i–v). The number −1, −2, +1, +2, ±1, ±2 represents negative cyan CPL, negative yellow CPL, positive cyan CPL, positive yellow CPL, cyan emission with no CPL activity, and yellow emission with no CPL activity, respectively.

Taking advantage of the distinct dynamic fluorescence and CPL feature of (**1**·**2**) HOF crystals, an advanced information encryption and decryption technology is conceptually demonstrated (**Figure** **6**). Two flower patterns made of five petals (i–v) are prepared by a mask‐assisted method. In the first pattern (Figure 6a,b), petals (i) and (ii) consist of ((+)‐car)_5.3_@(**1**·**2**) crystals, and petals (iii–v) are composed of ((−)‐car)_5.6_@(**1**·**2**) crystals. In addition, petals (ii), (iii), and (v) are covered with a layer of UV curable resin, followed by UV irradiation for 4 min, making them insensitive to thermo heating. In bright fields, all petals appear as yellow films and they all show similar cyan emission colors under UV light. These petals however can be distinguished by their CPL signals. Petals (i) and (ii) show (−)‐CPL and petals (iii–v) show (+)‐CPL (Figure S35, Supporting Information). This yields a five‐number code (−1 −1 +1 +1 +1), in which each number is sequentially determined by the optical state of the petal (i–v), and the number −1 and +1 stand for negative and positive cyan CPL, respectively. After heating for 24 h at 80 °C, the carvone molecules of petals (i) and (iv) are partially desorbed, leading to yellow CPLs. This results in a new code (−2 −1 +1 +2 +1), in which −2 and +2 stand for negative and positive yellow CPL, respectively. When the resulting pattern is further treated with acetone vapor, the original code (−1 −1 +1 +1 +1) is restored, as supported by the CPL measurements (Figure S35, Supporting Information). The pattern displayed in Figure 6c,d employs the CPL‐inactive DMF@(**1**·**2**) crystals to make some petals to create a series of more complicated codes. The original state is represented by code (−1 −1 ±1 ±1 +1), in which ±1 denotes cyan emission with no CPL activity. After heating at 80 °C for 1 or 24 h, a new code (−1 −1 ±1 ±2 +1) or (−1 −1 ±1 ±2 +1) is generated, in which ±2 represents yellow emission with no CPL activity. Again, this pattern can be restored to its original state by fumigation with acetone vapor, demonstrating the recyclable encryption capability of these HOF crystals.

To further understand the material's chiral memory properties, we conducted additional experiments to quantify the residual carvone content in the crystals after heating and subsequent acetone vapor treatment using ^1^H NMR analysis (Figure S36, Supporting Information). For the freshly prepared ((+)‐car)_5.3_@(**1**·**2**) crystals (petal (i) in Figure 6a,b) and ((−)‐car)_5.6_@(**1**·**2**) crystals (petal (iv) in Figure 6a,b), the process of heating at 80 °C for 24 h resulted in a composition of ((+)‐car)_2.0_@(**1**·**2**) and ((−)‐car)_2.3_@(**1**·**2**), respectively. After further treatment with acetone vapor, the carvone content was further reduced to afford ((+)‐car)_0.9_@(**1**·**2**) and ((−)‐car)_1.3_@(**1**·**2**), respectively. Similar carvone desorption and chiral memory properties were observed for the petals in Figure 6c,d after heating and subsequent acetone vapor treatment. These findings confirm that, although a significant portion of carvone is removed by heating and further acetone vapor treatment, the remaining small amount of carvone in the crystal is sufficient to induce CPL. This chiral memory effect is crucial for information storage and encryption.

## 3 Conclusion

In summary, this work presents an efficient strategy to achieve dynamic CPLs by using 2D HOFs. Highly luminescent 2D HOFs are obtained by the bicomponent assembly of a COOH‐functionalized linker and a pyridine‐functionalized AIE luminophore. By using a solvent exchange method, (+/−)‐carvone molecules are incorporated into the 1D channels of this HOF material. The obtained host‐guest chiral HOFs display distinct CPLs thanks to the efficient hydrogen bonding‐assisted chirality transfer from the chiral guests to the HOF structure. In particular, these chiral HOFs display dynamic CPLs with the CPL colors varying from cyan to yellow via a guest desorption‐triggered interlayer contraction mechanism. Interestingly, though the CPL colors are distinctly varied by the partial desorption of chiral solvent, the *g*_lum_ values remain essentially constant. This suggests that only the chiral solvent molecules bound to the HOF structures through hydrogen bonding directly influence the CPL magnitude of these materials. This information is critical for the future design of chiral host‐guest systems and the understanding of the underlying mechanism of chiroptical properties. This work showcases the great potential of HOFs to create smart materials with multiple switchable states distinguished by different chiroptical properties. In essence, the strategies of hydrogen bonding‐enhanced chirality transfer and guest desorption‐triggered interlayer contraction would be applicable to other flexible porous structures for the development of multifunctional and responsive materials.

## Conflict of Interest

The authors declare no conflict of interest.

## Supporting information



Supporting Information

Supplemental Video 1

## Data Availability

The data that support the findings of this study are available in the supplementary material of this article.
