# Supplementary material for: Achieving Dynamic Circularly Polarized Luminescence with 2D Hydrogen‐Bonded Organic Frameworks
Source: Adv Sci (Weinh). 2025 Mar 7;12(17):2500789. doi: 10.1002/advs.202500789 (PMC12061308; doi:10.1002/advs.202500789)
Supplement: Supplementary file 1 — Supporting Information [file ADVS-12-2500789-s002.pdf]

ADVANCED SCIENCE
Open Access

Supporting Information

for *Adv. Sci.*, DOI 10.1002/advs.202500789

Achieving Dynamic Circularly Polarized Luminescence with 2D Hydrogen-Bonded Organic Frameworks

Li Meng, Dian-Xue Ma, Zhong-Qiu Li, Tongling Liang, Zili Chen* and Yu-Wu Zhong**

Supporting Information for:

Achieving Dynamic Circularly Polarized Luminescence with 2D Hydrogen-Bonded Organic Frameworks

Li Meng,[a,b] Dian-Xue Ma,[a,c] Zhong-Qiu Li,*[a] Tongling Liang,[a] Zili Chen*[b] and Yu-Wu Zhong*[a,c]

[a]Beijing National Laboratory for Molecular Sciences, CAS Key Laboratory of Photochemistry, CAS Research/Education Center for Excellence in Molecular Sciences, Institute of Chemistry, Chinese Academy of Science, 2 Bei Yi Jie, Zhong Guan Cun, Haidian District, Beijing 100190, China

E-mail: lizhongqiu@iccas.ac.cn, zhongyuwu@iccas.ac.cn

[b]School of Chemistry and Life Resources, Renmin University of China, 59# Zhongguancun Street, Haidian District, Beijing 100872, China

E-mail: zilichen@ruc.edu.cn

[c]Institute of Molecular Engineering Plus, College of Chemistry, Fuzhou University, Fuzhou 350108, China

General information

Commercially available reagents and organic solvents were used as received without further purification. UV-visible (UV-vis) diffuse reflectance spectra was collected by a U-3900H UV-Vis spectrometer with $BaSO_4$ as the reference background. Photoluminescence (PL) spectra were measured at room temperature with an F-380 spectrofluorometer. Absolute fluorescence quantum yields (Φ_{FL}) were measured by a Hamamatsu Absolute PL Quantum Yield Spectrometer C11347 with an integrating sphere. Excited state emission lifetimes were recorded by a Hamamatsu C11367-11 instrument with single photon counting measurement. The morphology of HOF crystals was examined by SEM using Hitachi SU8010 instrument operating at 10 kV. TGA was carried out using the Pyris 1 TGA AIR instrument. N_2 and CO_2 sorption isotherms were performed on Micromeritics ASAP2020HD88 adsorption apparatus. Single-crystal X-ray analysis was performed on the Rigaku Saturn 724 diffractometer on a rotating anode at 173 K with Cu Kα radiation (1.54184 Å). The structure was solved by the direct method using SHELXS-97 and refined with Olex2. Powder X-ray diffractions (PXRD) were carried out on the Rigaku D/max-2500 instrument (Cu Kα, 1.54 Å). Fluorescence microscopy characterization was carried out using Olympus IX83 Inverted fluorescence microscope equipped with a spot-enhanced charge couple device (CCD, Diagnostic Instrument, Inc.) under UV irradiation (325-375 nm). CD spectra were recorded on a JASCO J-1700 spectrometer. CPL spectra were measured on a JASCO CPL-300 spectrometer.

Synthesis

Reagents and solvents were used as received. Compound tetra-(4-pyridylphenyl)ethylene (**2**)[1] and 5,5'-bis-(azanediyl)-oxalyldiisophthalic acid (**1**)[2] are known and were synthesized according to the reported literature.

Preparation of DMF@(1·2): Tetra-(4-pyridylphenyl)ethylene (**2**) (12.8 mg, 0.02 mmol) and 5,5'-bis-(azanediyl)-oxalyldiisophthalic acid (**1**) (21 mg, 0.05 mmol) were added to DMF (2 mL). The reaction mixture was heated in oil bath at 90 ℃ for 24 h, followed by cooling to room temperature slowly. Green crystals were collected by filtration and washed with cold DMF to give pure DMF@(**1·2**). Yields: 58.3%. ^{1}H NMR (400 MHz, DMSO-d_6): δ 11.26 (s, 2H), 8.72 (d, J = 1.5 Hz, 4H), 8.59 (d, J = 6.2 Hz, 8H), 8.27 (t, J = 1.6 Hz, 2H), 7.95 (s, 0.8H), 7.73 – 7.65 (m, 16H), 7.25 – 7.19 (m, 8H), 2.89 (s, 2.76H), 2.73 (s, 2.66H).

Preparation of ace@(1·2) and (1·2): The freshly prepared DMF@(**1·2**) crystals were immersed in acetone at 50 ℃ for 96 h. After that, the supernatant was removed, followed by the addition of a proper amount of fresh acetone. This exchange process was repeated for three times to give the crystal of ace@(**1·2**). The obtained crystal of ace@(**1·2**) was further degassed under vacuum at 60 ℃ for 6 h to afford the solvent-free sample of (**1·2**) as yellow crystals in 79.7% yield. ^{1}H NMR (400 MHz, DMSO-d_6): δ 11.24 (s, 2H), 8.71 (d, J = 1.5 Hz, 4H), 8.66 – 8.53 (m, 8H), 8.27 (t, J = 1.6 Hz, 2H), 7.78 – 7.58 (m, 16H), 7.27 – 7.17 (m, 8H). ESI-HRMS (P) caclcd for $C_{64}H_{33}N_4$ [**2** + H]$^+$: 641.2698; found: 641.2670. ESI-HRMS (N) caclcd for $C_{18}H_{11}N_2O_{10}$ [**1** − H]$^-$: 415.0417; found: 415.0419.

Preparation of (+)-car@(1·2) and (−)-car@(1·2): The crystals of (+)-car@(**1·2**) and

(−)-car@(**1·2**) were prepared through a similar exchange procedure by immersing ace@(**1·2**) crystals into (+)- or (−)-carvone at 50 ℃ for 96 h. The obtained cyan crystals were collected by filtration and washed with (+)- or (−)-carvone to give the target samples in 61.4% and 63.5% yield. ^{1}H NMR (400 MHz, DMSO-d_6): δ 11.24 (s, 2H), 8.71 (s, 4H), 8.59 (d, J = 5.3 Hz, 8H), 8.27 (s, 2H), 7.78 – 7.61 (m, 16H), 7.27 – 7.19 (m, 8H), 6.85 (s, 3.18H), 4.76 (d, J = 8.7 Hz, 6.78H), 2.68 – 2.62 (m, 3.40H), 2.41 – 2.25 (m, 12.78H), 1.76 – 1.60 (m, 18.94H). ESI-HRMS (P) caclcd for $C_{64}H_{33}N_4$ [**2** + H]$^+$: 641.2697; found: 641.2670. ESI-HRMS (N) caclcd for $C_{18}H_{11}N_2O_{10}$ [**1** – H]$^-$: 415.0419; found: 415.0419. ESI-HRMS (P) caclcd for $C_{10}H_{14}NaO$ [carvone + Na]$^+$: 173.0937; found: 173.0937.

Preparation of (+)-cin@(1·2) and (−)-cin@(1·2): (+)-cin@(**1·2**) and (−)-cin@(**1·2**) were prepared through a similar exchange procedure by immersing ace@(**1·2**) into (+)- or (−)-cinene at 50 ℃ for 96 h. ^{1}H NMR (400 MHz, DMSO-d_6): δ 11.23 (s, 2H), 8.71 (s, 4H), 8.59 (d, J = 5.9 Hz, 8H), 8.27 (s, 2H), 7.77 – 7.59 (m, 16H), 7.31 – 7.15 (m, 8H), 5.37 (s, 1.42H), 4.75 – 4.65 (m, 2.85H), 2.10 – 1.89 (m, 6.54H), 1.74 – 1.59 (m, 10.22H), 1.42 – 1.36 (m, 1.79H).

Table S1. Crystallographic data and parameters.

HOF	DMF@(**1·2**)	ace@(**1·2**)	(+)-car@(**1·2**)[a]
CCDC number	2305493	2305496	2400869
empirical formula	$C_{70}H_{58}N_8O_{12}$	$C_{70}H_{56}N_6O_{12}$	$C_{336}H_{288}N_{24}O_{48}$
formula weight	1203.24	1173.20	5429.89
Temperature (K)	170.00(16)	170.00(10)	169.99(10)
crystal system	orthorhombic	orthorhombic	triclinic
Space group	*P*bcn	*P*bcn	*P*1
a (Å)	36.7823(4)	36.8884(8)	16.3422(5)
b (Å)	16.0496(3)	15.7788(4)	24.9025(4)
c (Å)	33.9771(4)	33.8166(7)	25.2142(3)
α (°)	90	90	85.1530(10)
β (°)	90	90	85.425(2)
γ (°)	90	90	83.699(2)
V ($Å^3$)	20058.1(5)	19683.1(8)	10137.0(4)
Z value	8	8	1
Density (g/cm^3)	0.797	0.792	0.889
R1 (final)	0.0774	0.1089	0.1049
wR2 (final)	0.2524	0.3115	0.2841
R1 (all)	0.0956	0.1750	0.1402
wR2 (all)	0.2717	0.3628	0.3240

[a]The single-crystal quality is relatively low due to the disordered arrangement of the carvone guests, coupled with the issues of twinning and structural disorder. As a result, the crystal exhibits poor diffraction at high angles. See Figure S11.

Table S2. Photophysics data of microcrystals.

HOF	$\lambda_{em,max}$/nm	τ/ns	Φ^{a}_{FL}/%	$g_{lum}{}^{b}/(10^{-3})$
(**1·2**)	543	4.65	92.5	--
DMF_2@(**1·2**)	522	3.09	28.4	--
((+)-car)$_{5.5}$@(**1·2**)	495	2.64	28.1	−5.10
((−)-car)$_{4.7}$@(**1·2**)	495	2.62	28.2	4.80
((+)-car)$_{3.2}$@(**1·2**)	510	3.17	49.7	−5.98
((−)-car)$_{3.3}$@(**1·2**)	510	2.98	47.8	5.09
((+)-car)$_{1.9}$@(**1·2**)	533	3.65	78.8	−6.91
((−)-car)$_{2.5}$@(**1·2**)	534	3.66	79.4	6.40
((+)-car)$_{1.3}$@(**1·2**)	540	2.43	54.2	−5.27
((−)-car)$_{1.1}$@(**1·2**)	541	2.79	50.8	6.93
((+)-car)$_{0.3}$@(**1·2**)	545	2.90	51.3	−4.18
((−)-car)$_{0.5}$@(**1·2**)	545	2.56	51.7	4.60
((+)-car)$_{<0.01}$@(**1·2**)	545	2.76	38.9	−1.48
((−)-car)$_{<0.01}$@(**1·2**)	545	2.81	38.7	1.53

[a]Absolute fluorescence quantum yields. [b]Luminescence dissymmetry is defined as $2(I_L\text{-}I_R)/(I_L+I_R)$, where I_L and I_R represent the intensity of left- and right-handed CPL, respectively. The values were calculated according to g_{lum} = [ellipticity/(32980/ln10)]/total fluorescence intensity at the CPL extremum.[3] The excitation wavelength is 365 nm for all measurements.

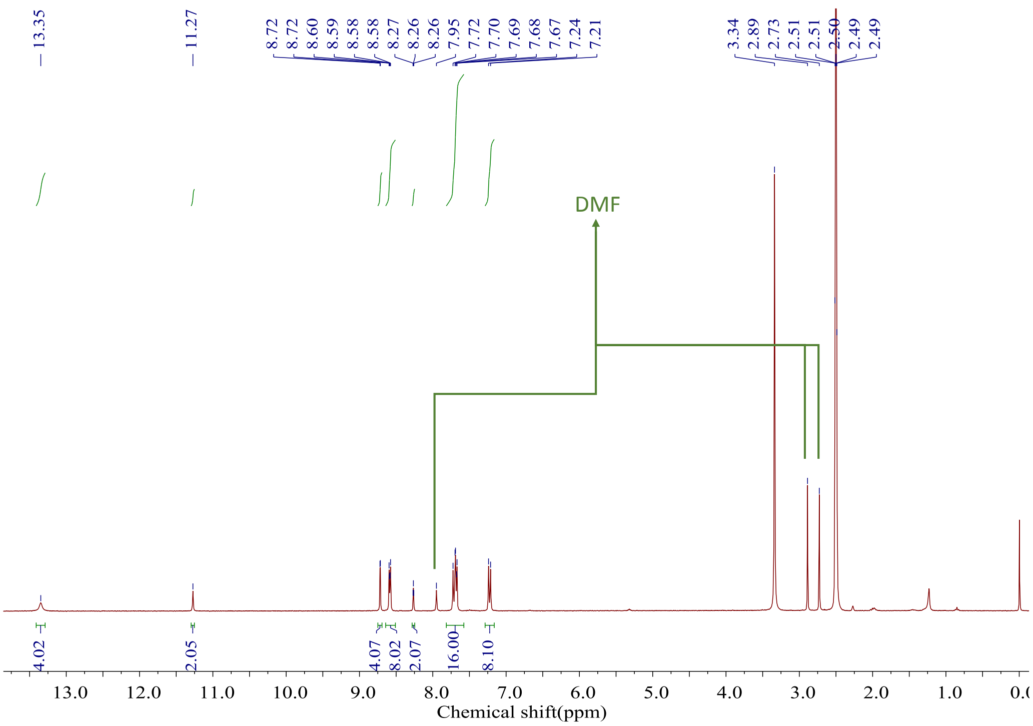

Figure S1. ^{1}H NMR spectrum of DMF@(**1** **2**) in DMSO-d_6.

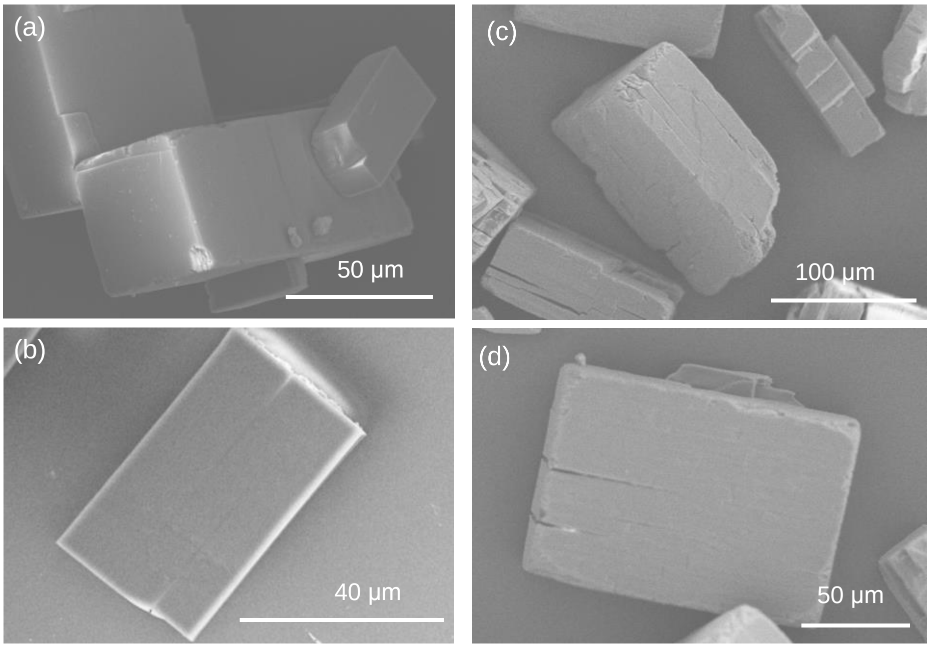

Figure S2. SEM images of crystals of (a, b) $(DMF)_2$@(**1** **2**), (c, d) solvent-free (**1** **2**).

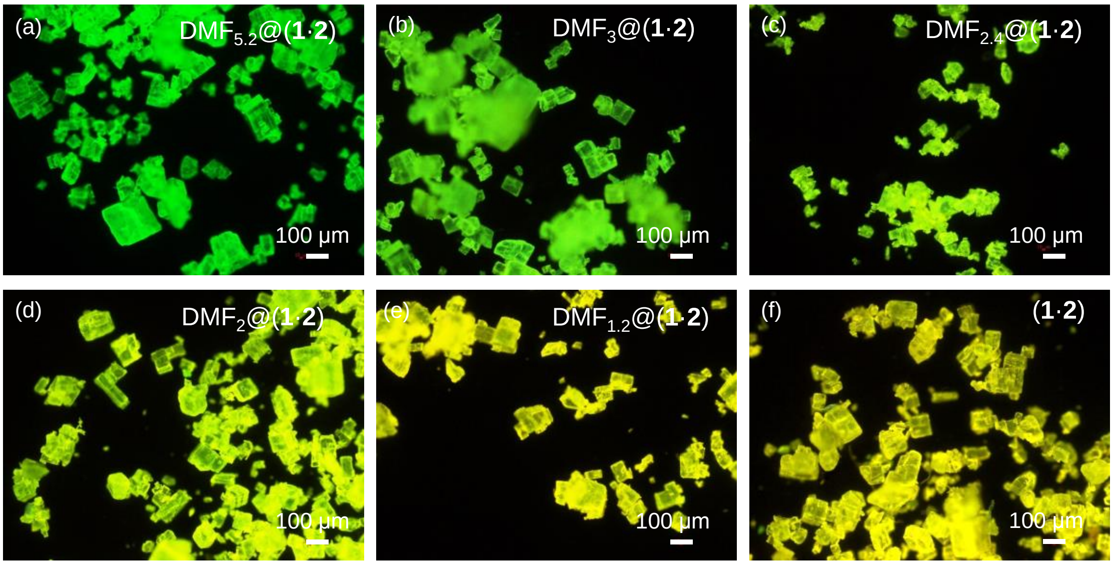

Figure S3. Fluorescence microscopy images of DMF@(**1**·**2**) containing different contents of DMF.

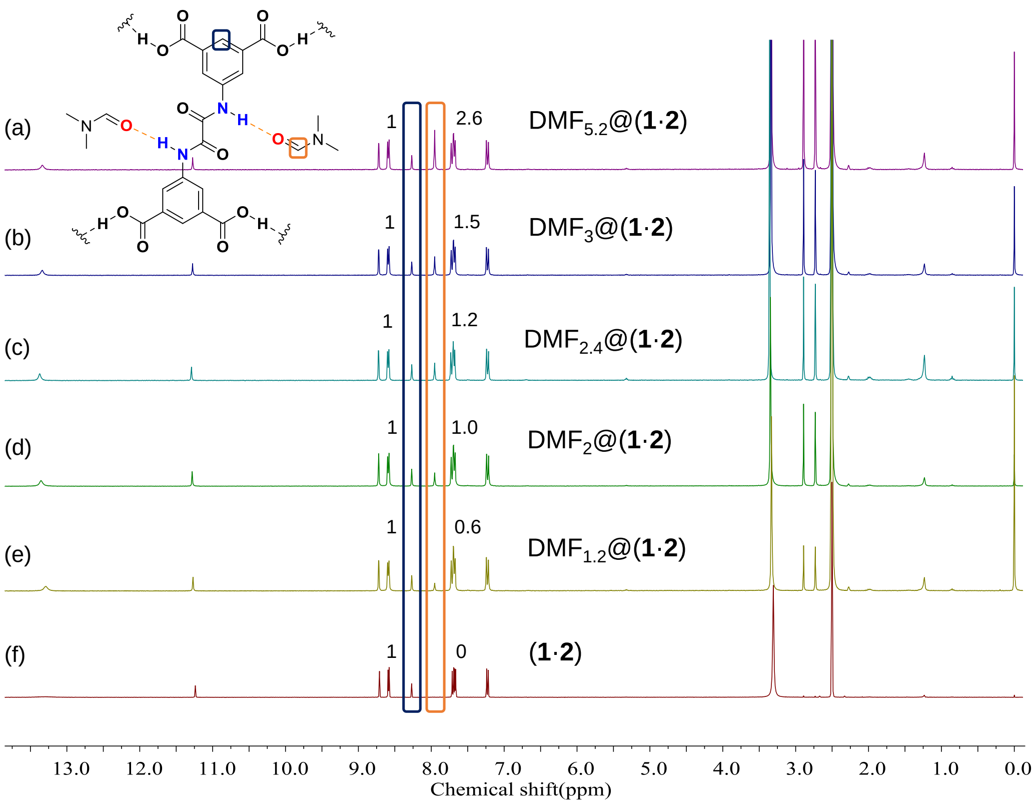

Figure S4. ^{1}H NMR spectra in DMSO-d_6 of DMF@(**1**·**2**) aftering storing in air at room temperature for different period. (a) 0 day, (b) 5 days, (c) 15 days, (d) 1 month, (e) 3 months, (f) 6 months. The navy-blue and orange rectangle indicates one phenyl proton of the HOF framework and the DMF aldehyde proton, respectively.

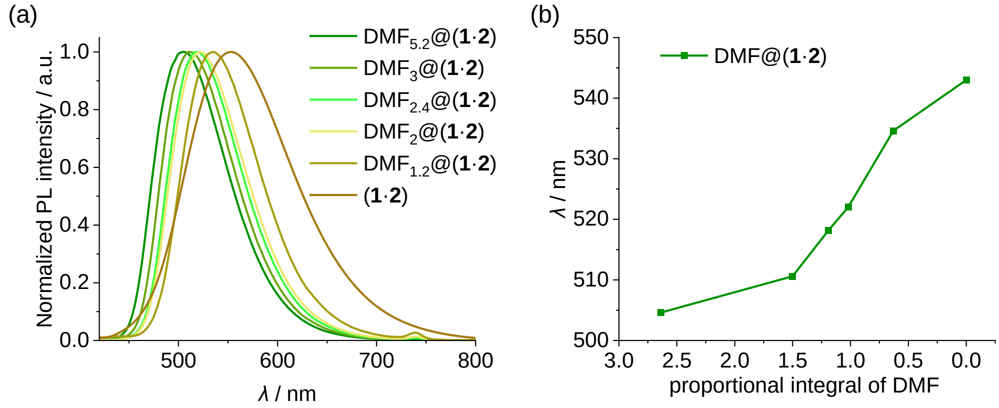

Figure S5. (a) Photoluminescence (PL) spectra and (b) plot of proportional integral of DMF vs maximum emission wavelength of DMF@(**1** **2**).

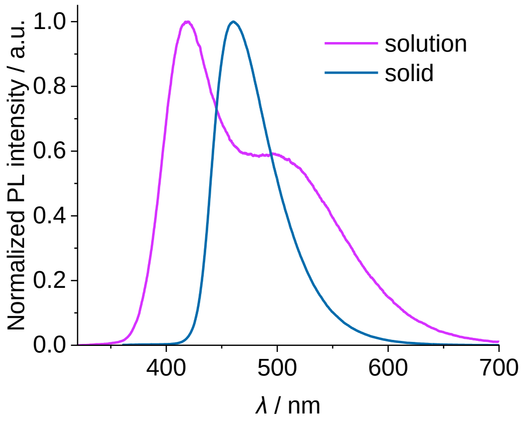

Figure S6. PL spectra of **2** in DMF (1 ×10^{-5} M) (λ_{ex} = 300 nm) and solid state (λ_{ex} = 340 nm).

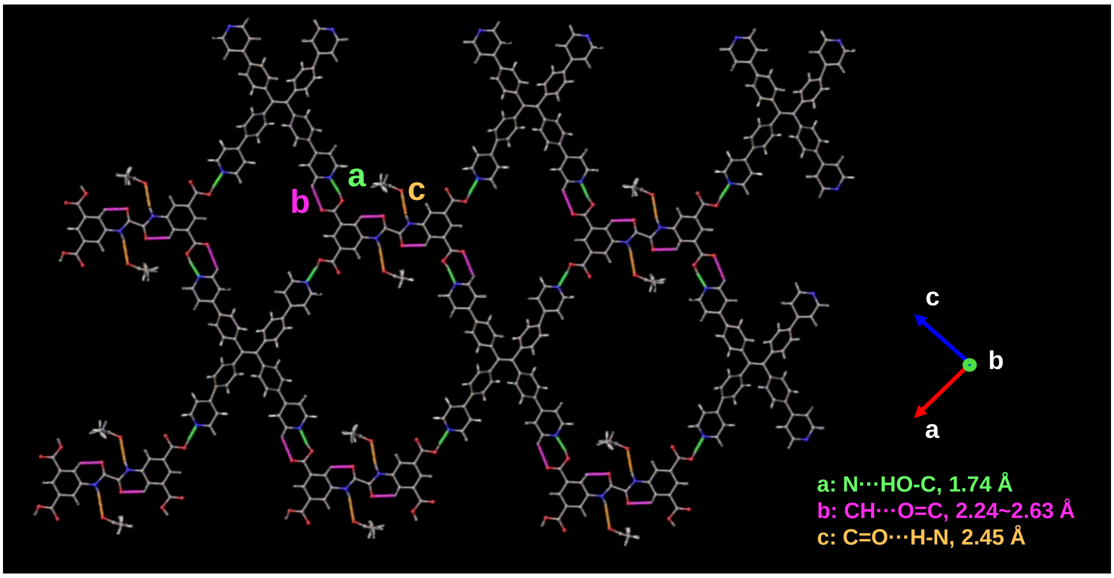

Figure S7. Single-layer crystal structure of ace@(**1** **2**) along the crystallographic *b* axis, with major non-covalent interactions being indicated.

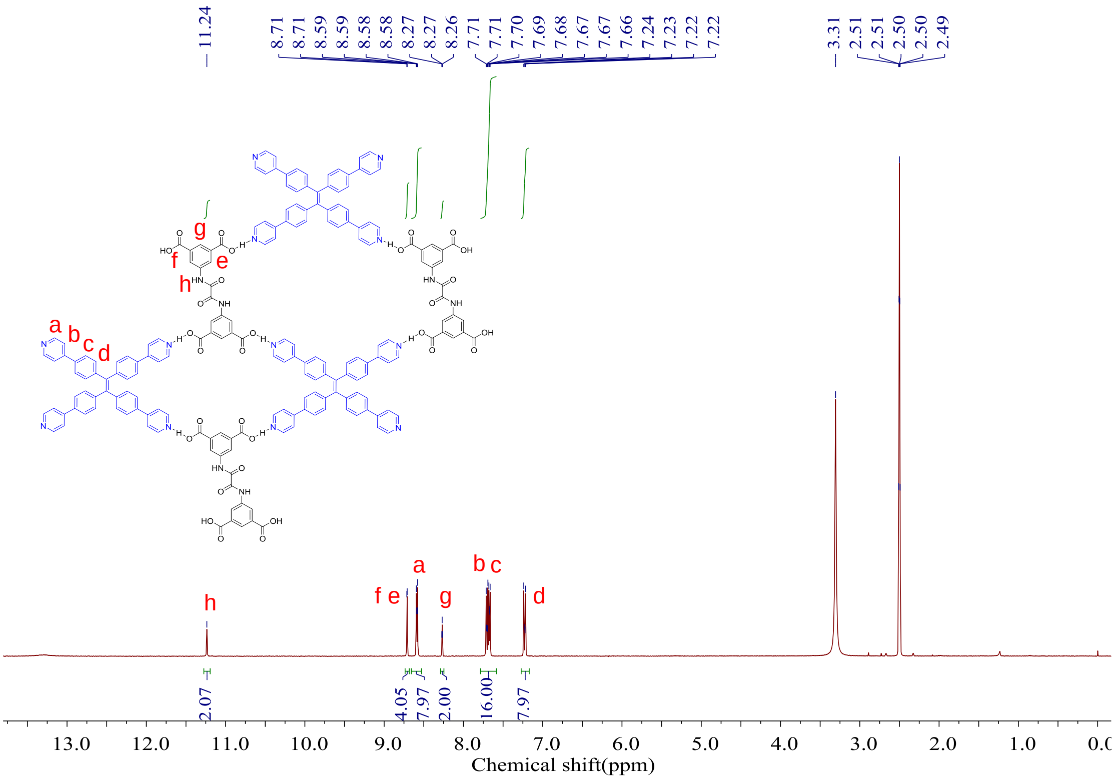

Figure S8. ^{1}H NMR spectra of (**1** **2**) in DMSO-d_6.

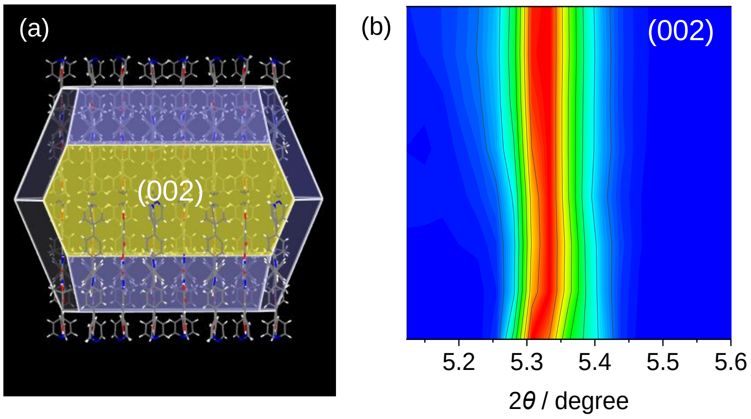

Figure S9. (a) Simulated growth morphology of (**1** **2**) viewed from the (002) plane. (b) Evolution of the normalized intensity of the (002) peak of ace@(**1** **2**) upon the desorption of acetone.

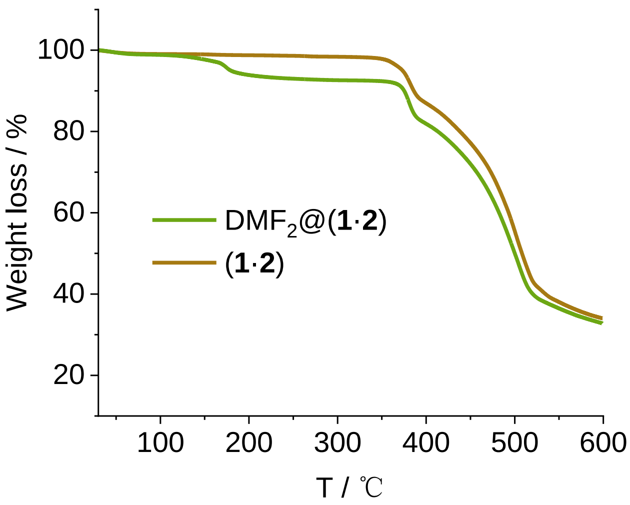

Figure S10. TGA curves of DMF$_2$@(**1 2**) and (**1 2**).

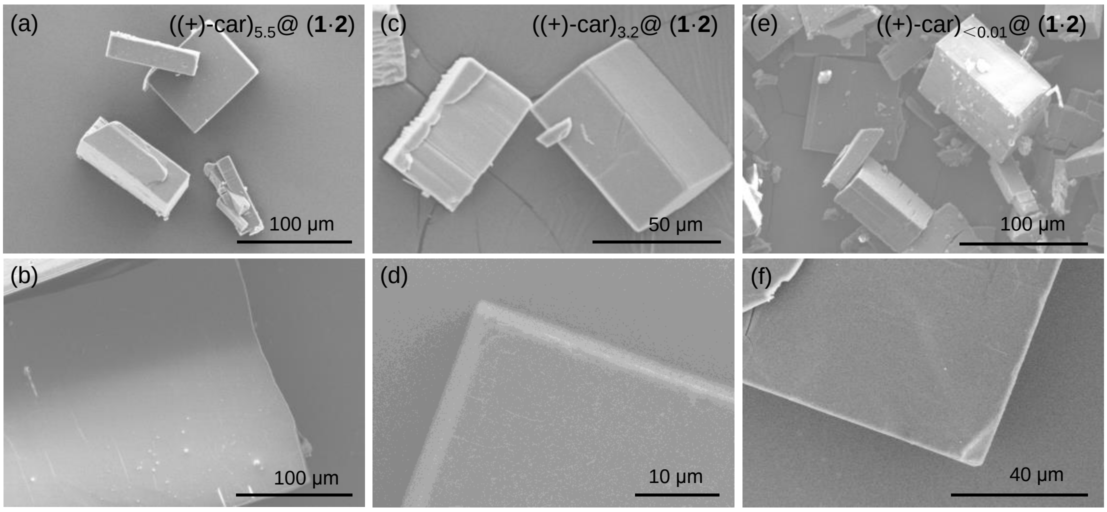

Figure S11. SEM images of crystals of (a, b) ((+)-car)$_{5.5}$@(**1 2**), (c, d) ((+)-car)$_{3.2}$@(**1 2**) and (e, f) ((+)-car)$_{<0.01}$@(**1 2**).

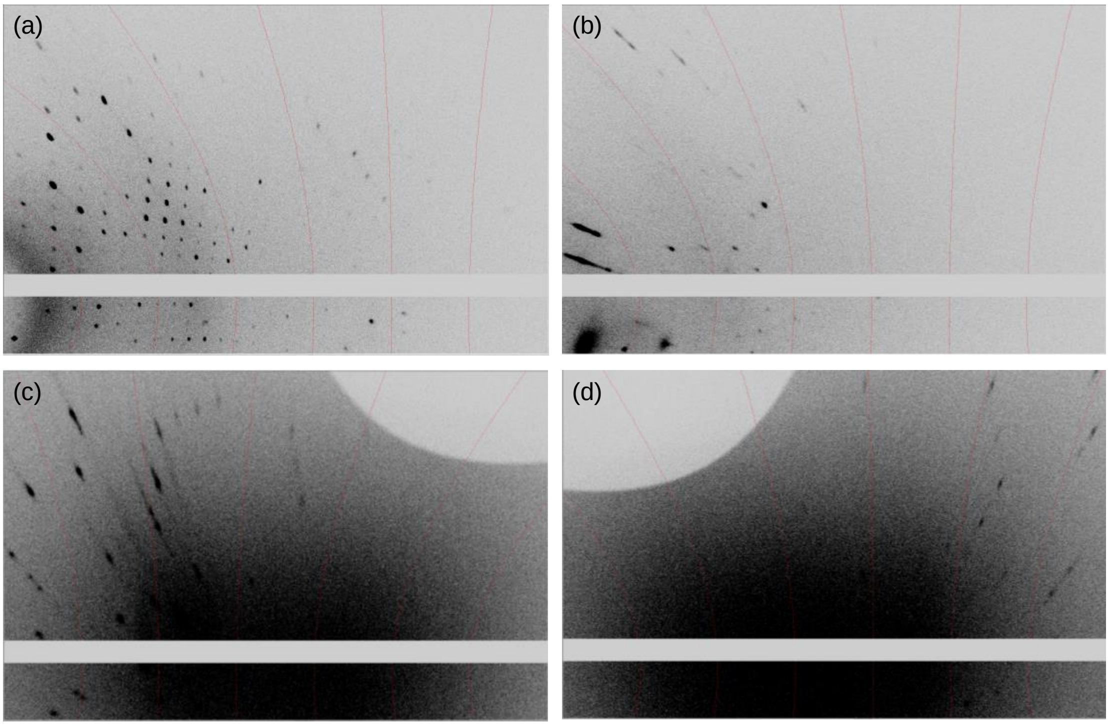

Figure S12. Print-screen images of the single frames of the (+)-car@(**1**·**2**) crystal recorded at (a, b) low diffraction angles and (c, d) high diffraction angles.

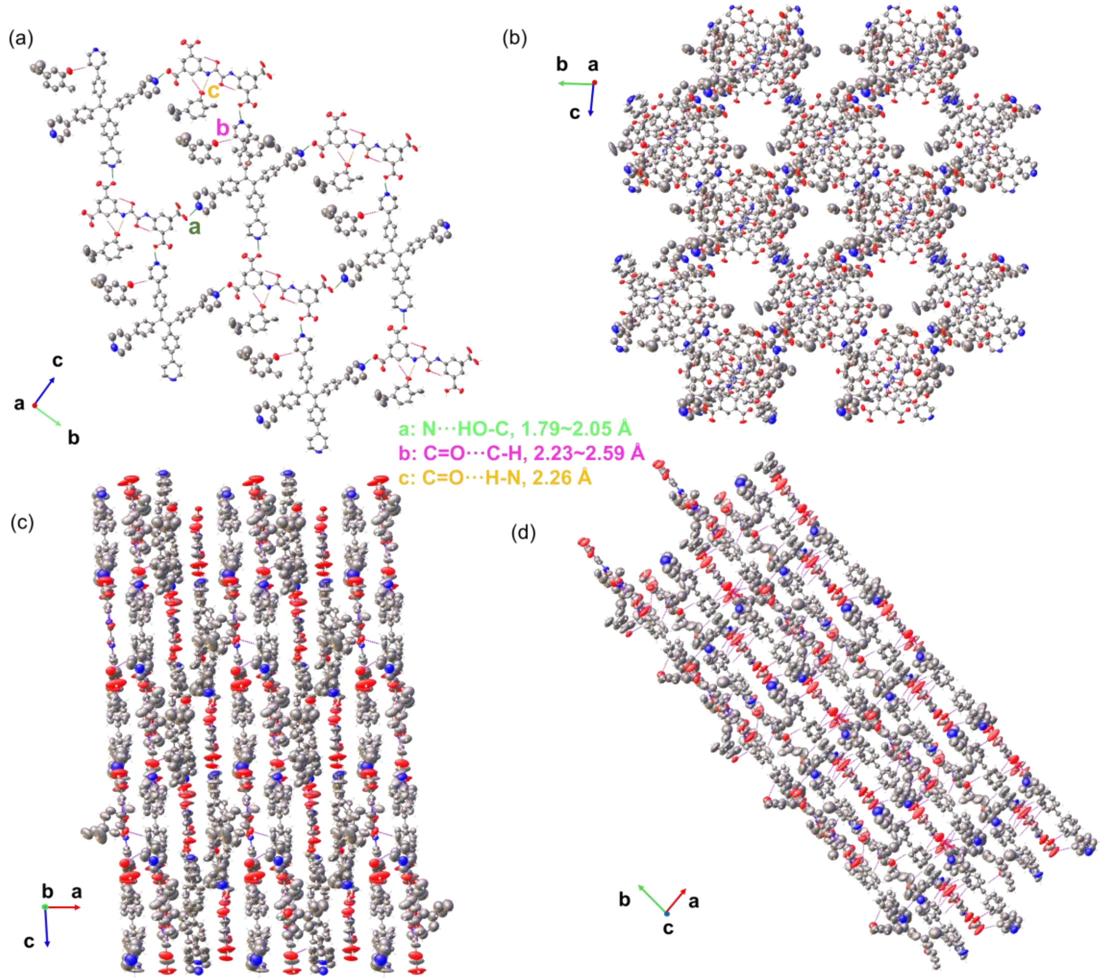

Figure S13. (a) Thermal ellipsoid plots at 30% probability of the single-crystal structures of (+)-car@(**1 2**), with major non-covalent interactions being indicated. (b-d) crystal packing of (+)-car@(**1 2**) along the crystallographic (b) *a* axis, (c) *b* axis and (d) *c* axis.

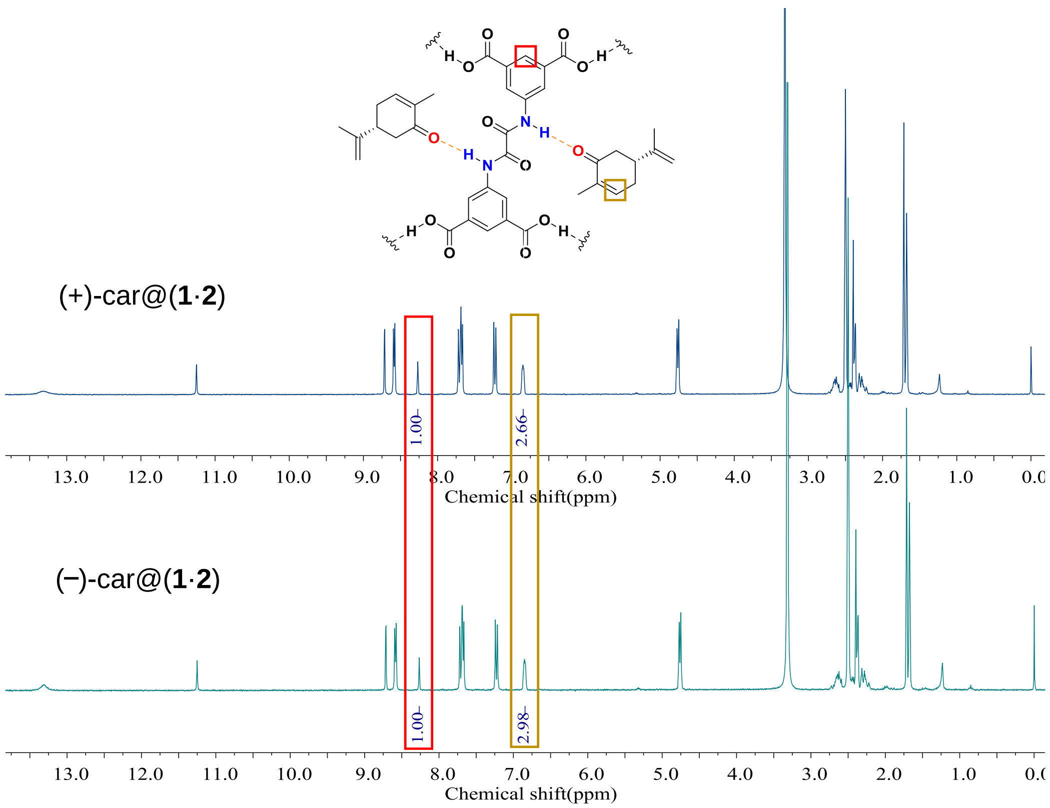

Figure S14. ^{1}H NMR spectra of freshly prepared (+)-car@(**1 2**) and (−)-car@(**1 2**) in DMSO-d_6. The red and dark-yellow rectangle indicates one phenyl proton of the HOF framework and one olefin proton of carvone, respectively.

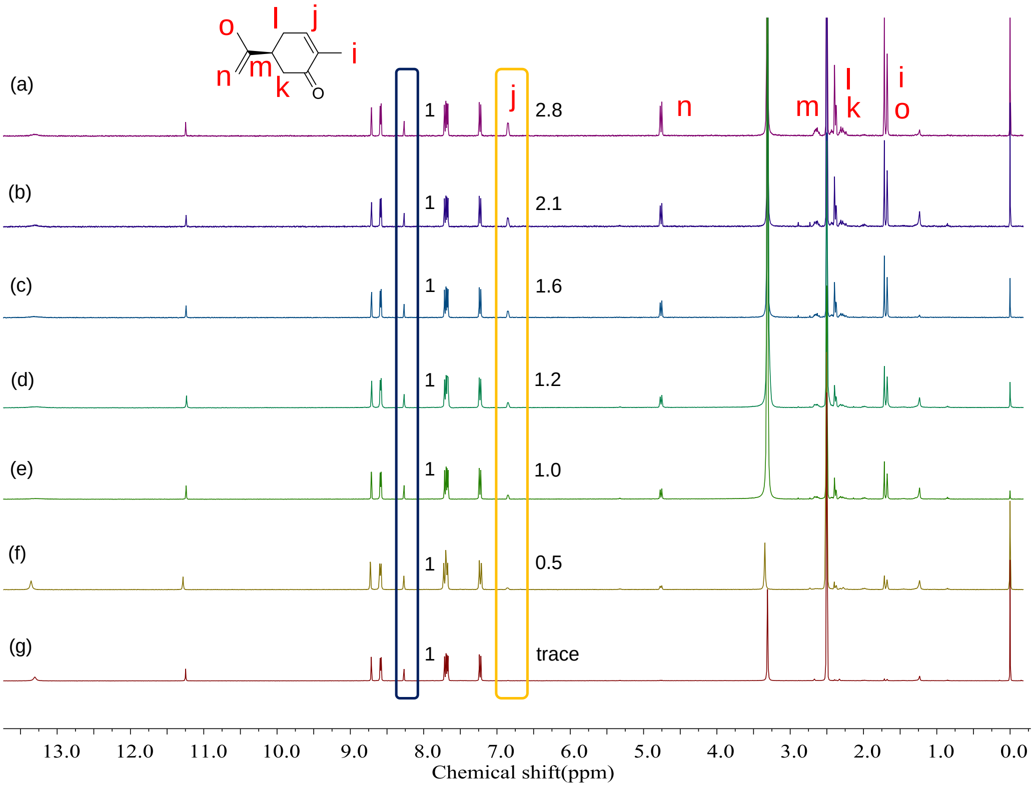

Figure S15. ^{1}H NMR spectra in DMSO-d_6 of (+)-car@(**1**·**2**) after storing in air at room temperature for different period. (a) 0 day, (b) 5 days, (c) 15 days, (d) 1 month, (e) 3 months, (f) 6 months, (g) 12 months. The navy-blue and orange rectangle indicates one phenyl proton of the HOF framework and one olefin proton of carvone, respectively.

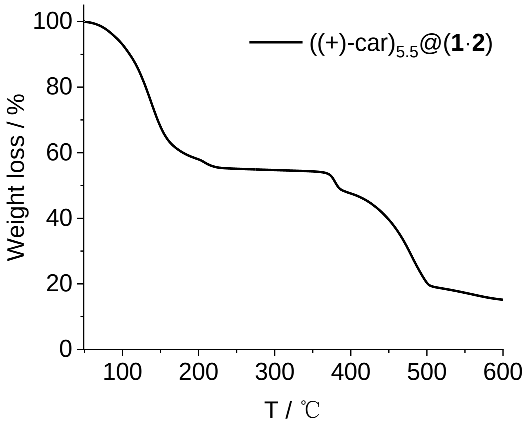

Figure S16. TGA curve of ((+)-car)$_{5.5}$@(**1**·**2**).

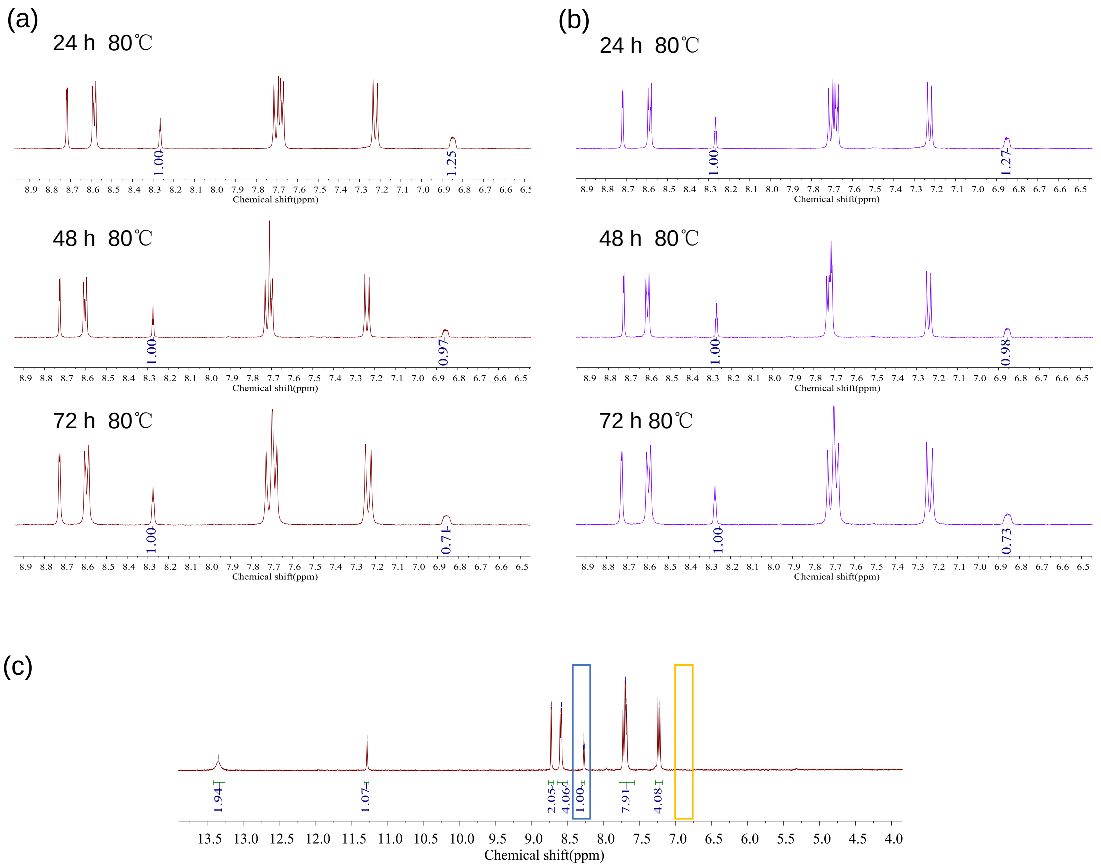

Figure S17. (a,b) ^{1}H NMR spectra in DMSO-d_6 of (a) (+)-car@(**1 2**) and (b) (−)-car@(**1 2**) after storing in an open environment at 80 ℃ for different hours. (c) ^{1}H NMR spectrum of ((+)-car)$_{<0.01}$@(**1 2**) obtained by heating (+)-car@(**1 2**) at 80 ^{o}C under vacuum. The navy-blue rectangle indicates one phenyl proton of the HOF framework. The orange rectangle is free of any signal, suggesting that the conent of (+)-carvone within this ((+)-car)$_{<0.01}$@(**1 2**) HOF structure, if any, is negligible.

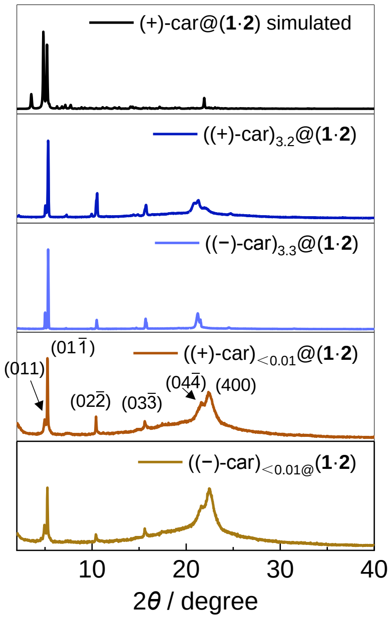

Figure S18. PXRD patterns of ((+)-car)$_{3.2}$@(**1**·**2**), ((−)-car)$_{3.3}$@(**1**·**2**), ((+)-car)$_{<0.01}$@(**1**·**2**) and ((−)-car)$_{<0.01}$@(**1**·**2**). The simulated patterns of (+)-car@(**1**·**2**) are included on the top of the figure for the purpose of comparison.

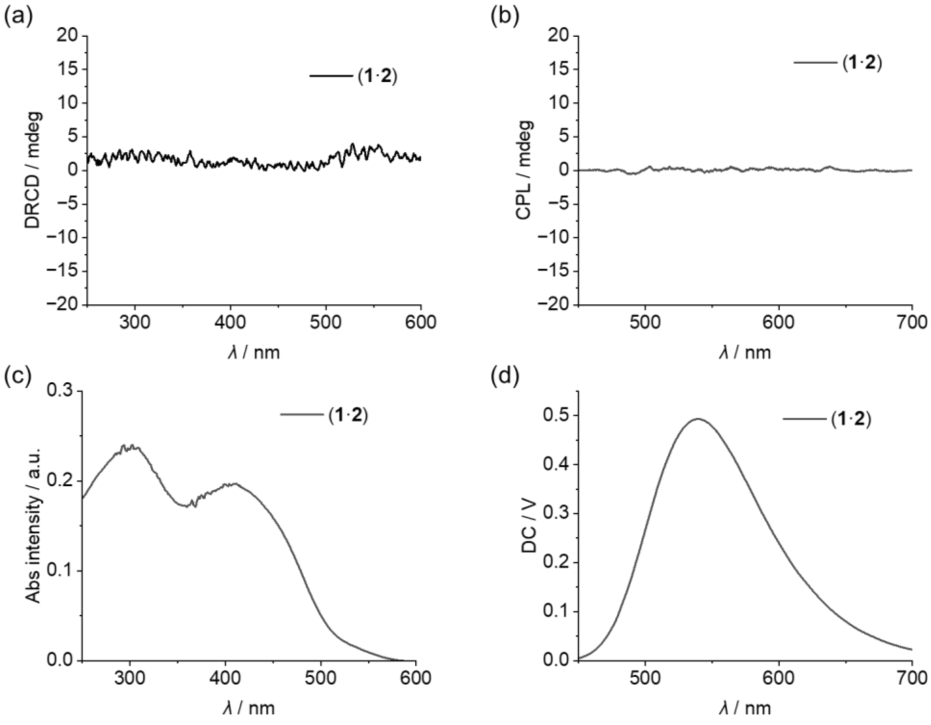

Figure S19. (a) DRCD, (b) CPL, (c) absorption and (d) emission spectra of the solvent-free crystal of (**1**·**2**).

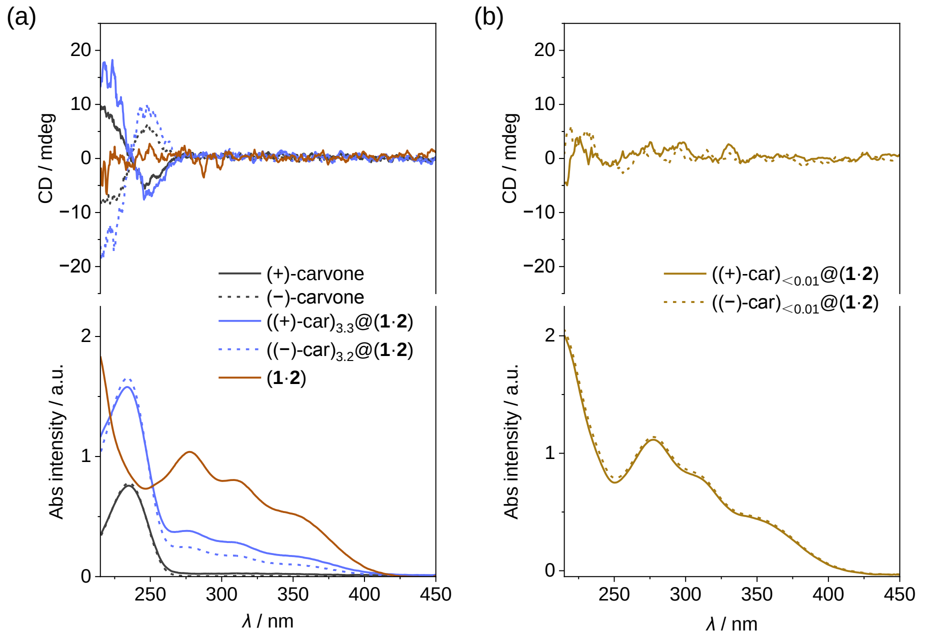

Figure S20. CD (upper) and UV-vis absorption (below) spectra of the solution of (a) (+)-carvone, (−)-carvone, ((+)-car)$_{3.2}$@(**1·2**), ((−)-car)$_{3.2}$@(**1·2**) and (**1·2**) and (b) ((+)-car)$_{<0.01}$@(**1·2**) and ((−)-car)$_{<0.01}$@(**1·2**) in EtOH. The solution of carvone has a concentration of 6 × 10^{-5} M.

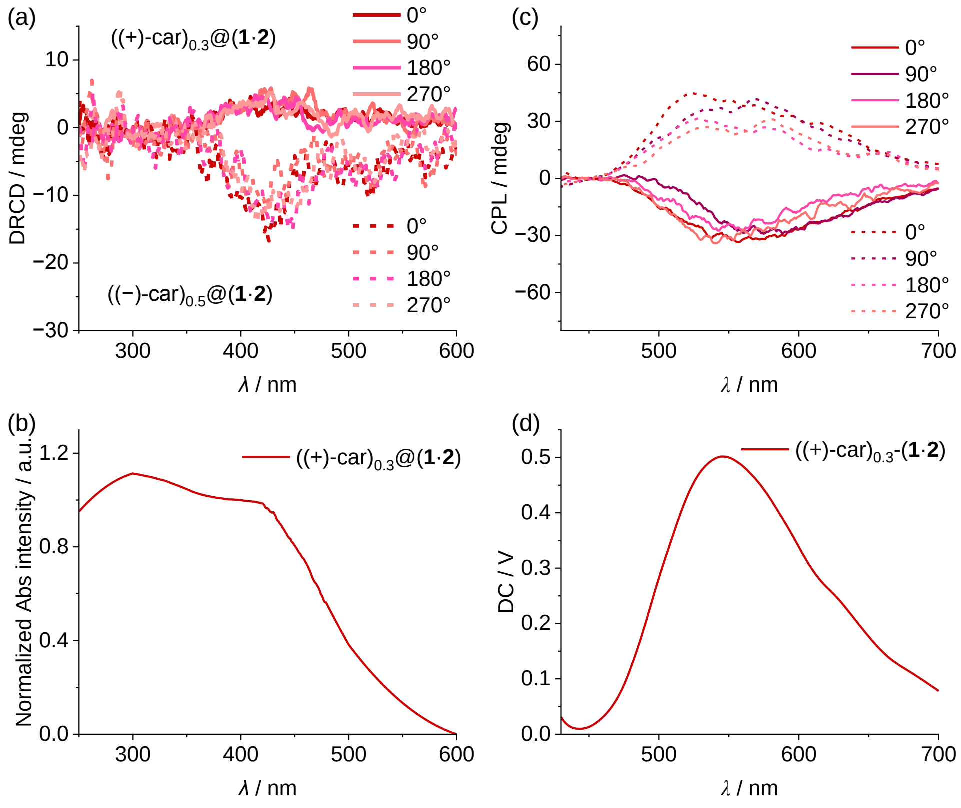

Figure S21. (a) DRCD, (b) absorption, (c) CPL and (d) emission spectra measured at different substrate angles from 0 ° to 270 ° of ((+)-car)$_{0.3}$@(**1·2**), ((−)-car)$_{0.5}$@(**1·2**).

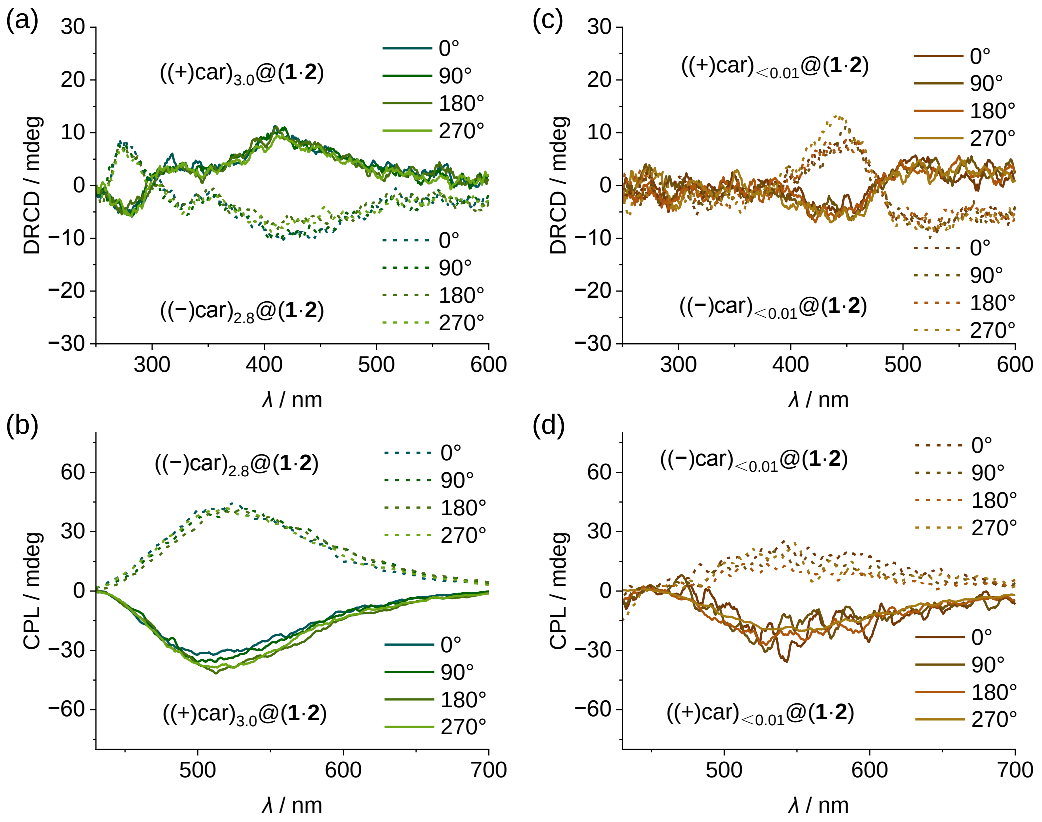

Figure S22. (a, c) CD and (b, d) CPL spectra of (a, b) ((+)-car)$_{3.0}$@(**1 2**) and ((−)-car)$_{2.8}$@(**1 2**) and (c, d) ((+)-car)$_{<0.01}$@(**1 2**) and ((−)-car) $_{<0.01}$@(**1 2**) after storing in a sealed tube at rt for 1 year.

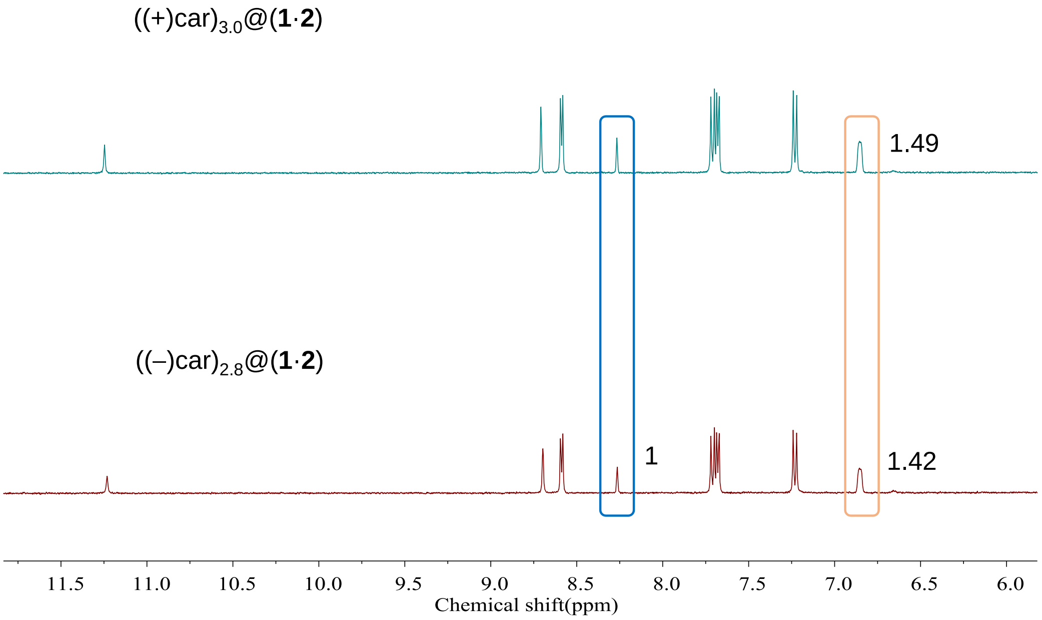

Figure S23. ^{1}H NMR spectrum of ((+)-car)$_{3.0}$@(**1**·**2**) and ((−)-car)$_{2.8}$@(**1**·**2**) after storing in a sealed container at rt for 1 year (DMSO-d_6). The navy-blue and orange rectangle indicates one phenyl proton of the HOF framework and one olefin proton of carvone, respectively.

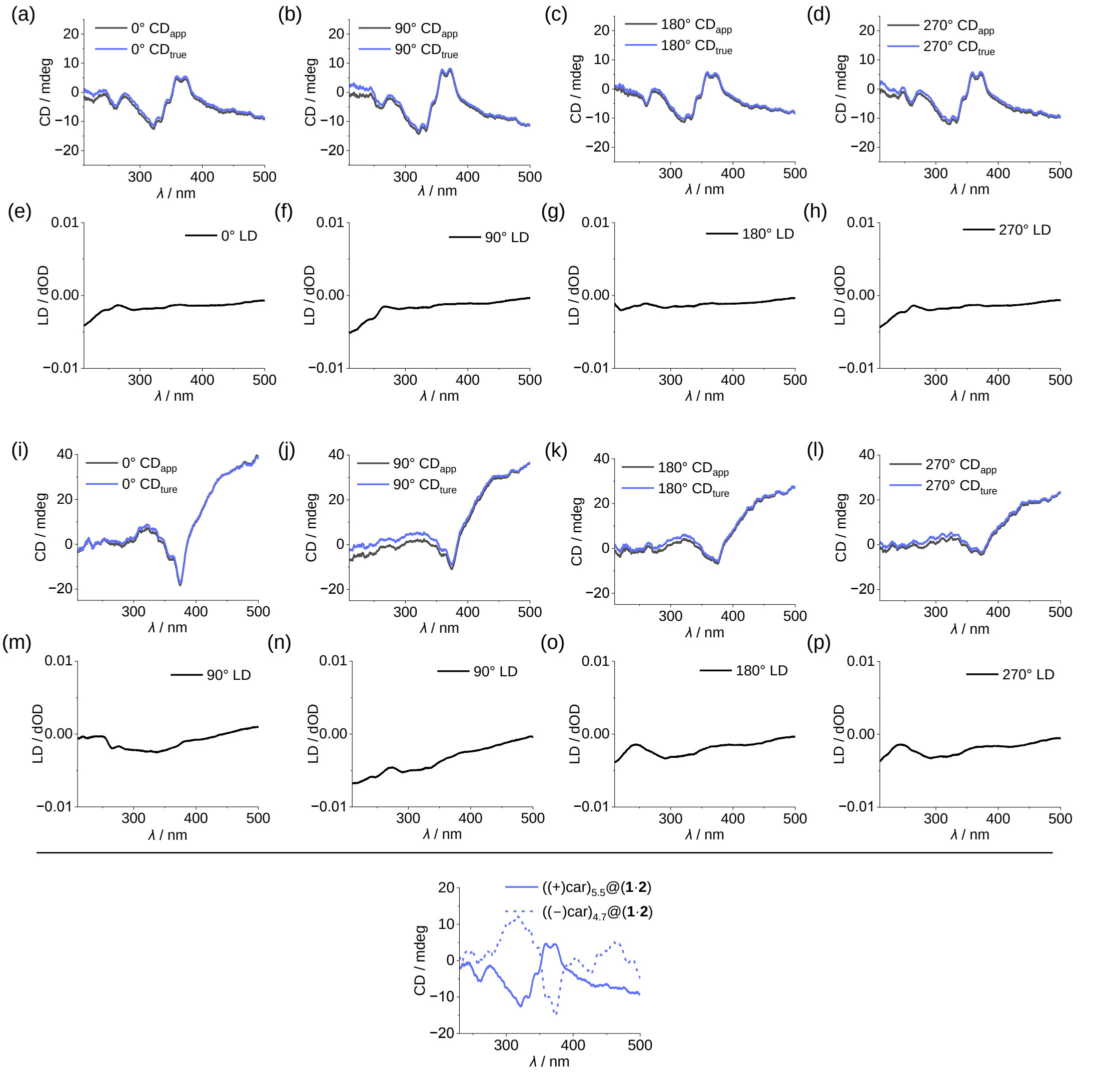

Figure S24. CD (upper) and relevant LD (below) spectra measured at different substrate angles from 0 °to 270 °of (a-h) ((+)-car)$_{5.5}$@(**1 2**) and (i-p) ((−)-car)$_{4.7}$@(**1 2**) microcrystals. CD spectra are measured at the transmission mode. CD$_{app}$ denotes the observed CD signal, CD$_{true}$ is the true CD signal, and LD represents the relevant linear dichromism signal. CD$_{true}$ were calculated according to CD$_{ture}$ = CD$_{app}$ – LD ×0.02.[3]

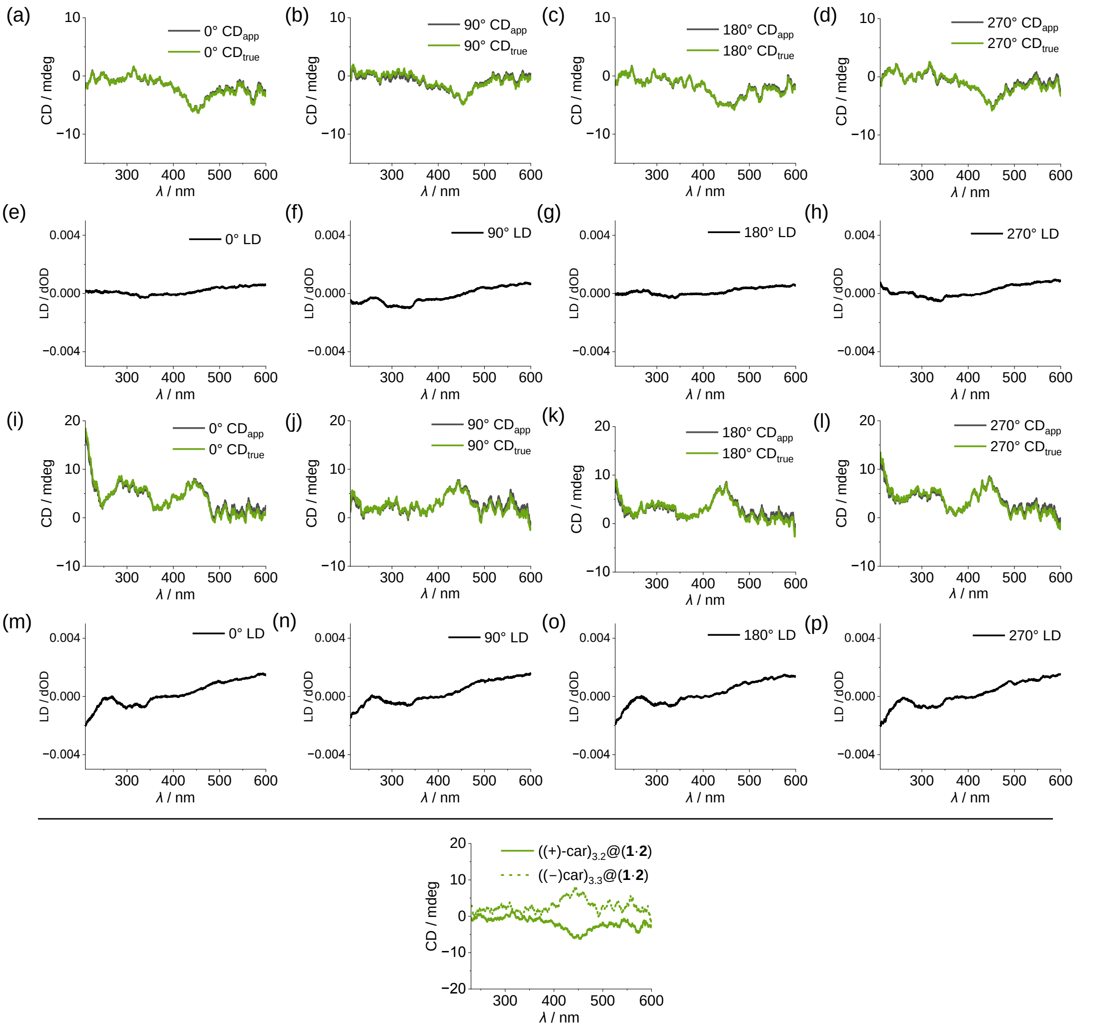

Figure S25. CD (upper) and relevant LD (below) spectra measured at different substrate angles from 0 °to 270 °of (a-h) ((+)-car)$_{3.2}$@(**1 2**) and (i-p) ((−)-car)$_{3.3}$@(**1 2**) microcrystals. CD spectra are measured at the transmission mode.

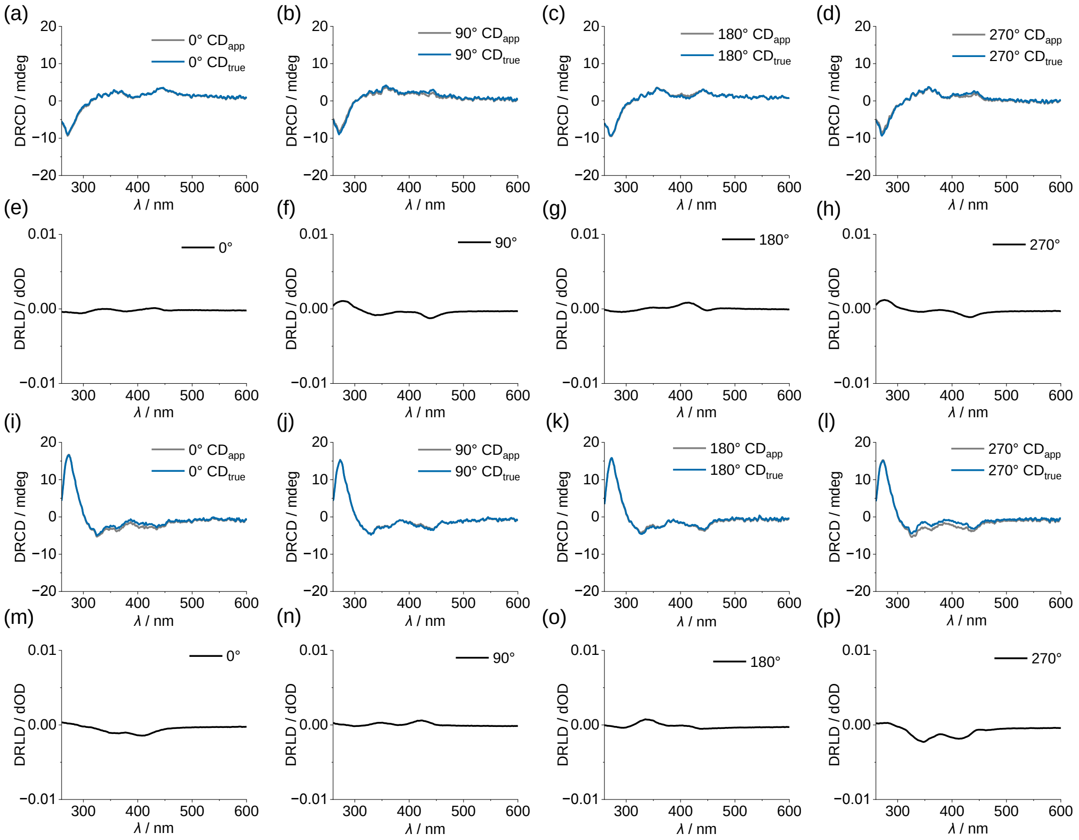

Figure S26. DRCD (upper) and relevant DRLD (below) spectra measured at different substrate angles from 0 ° to 270 ° of (a-h) ((+)-car)$_{5.5}$@(**1 2**) and (i-p) ((−)-car)$_{4.7}$@(**1 2**) microcrystals.[3]

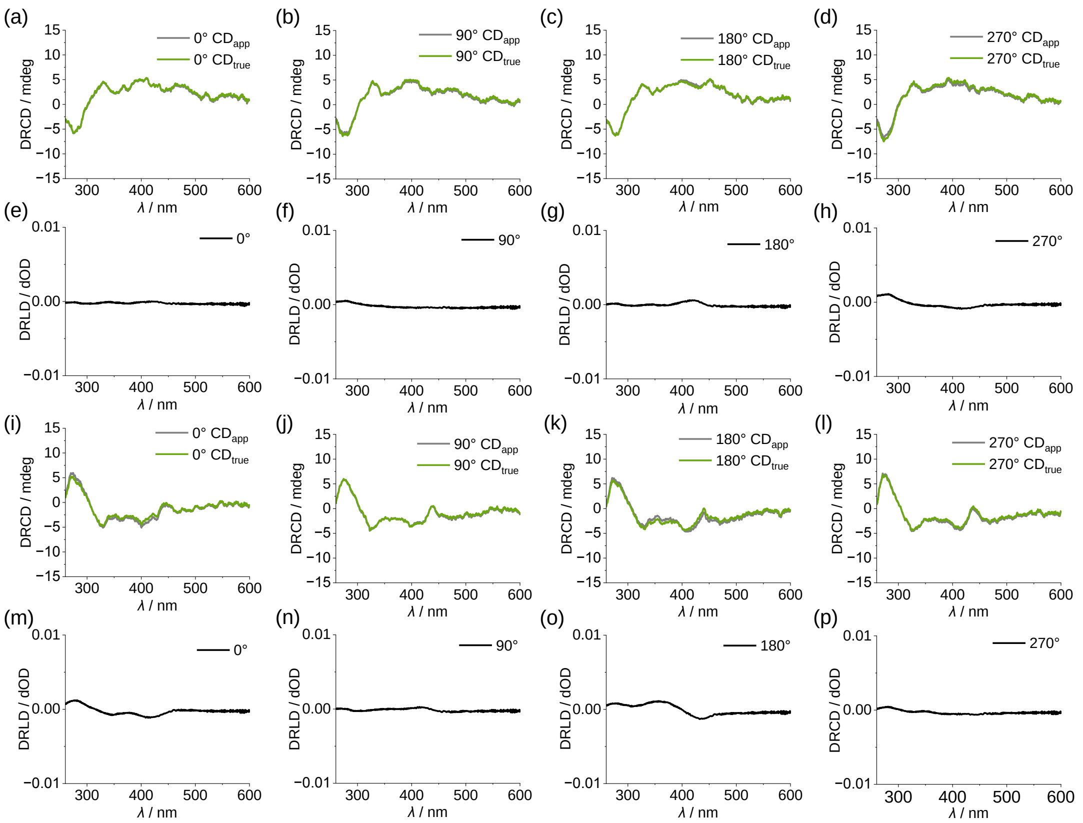

Figure S27. DRCD (upper) and relevant DRLD (below) spectra measured at different substrate angles from 0 ° to 270 ° of (a-h) ((+)-car)$_{3.2}$@(**1 2**) and (i-p) ((−)-car)$_{3.3}$@(**1 2**) microcrystals.

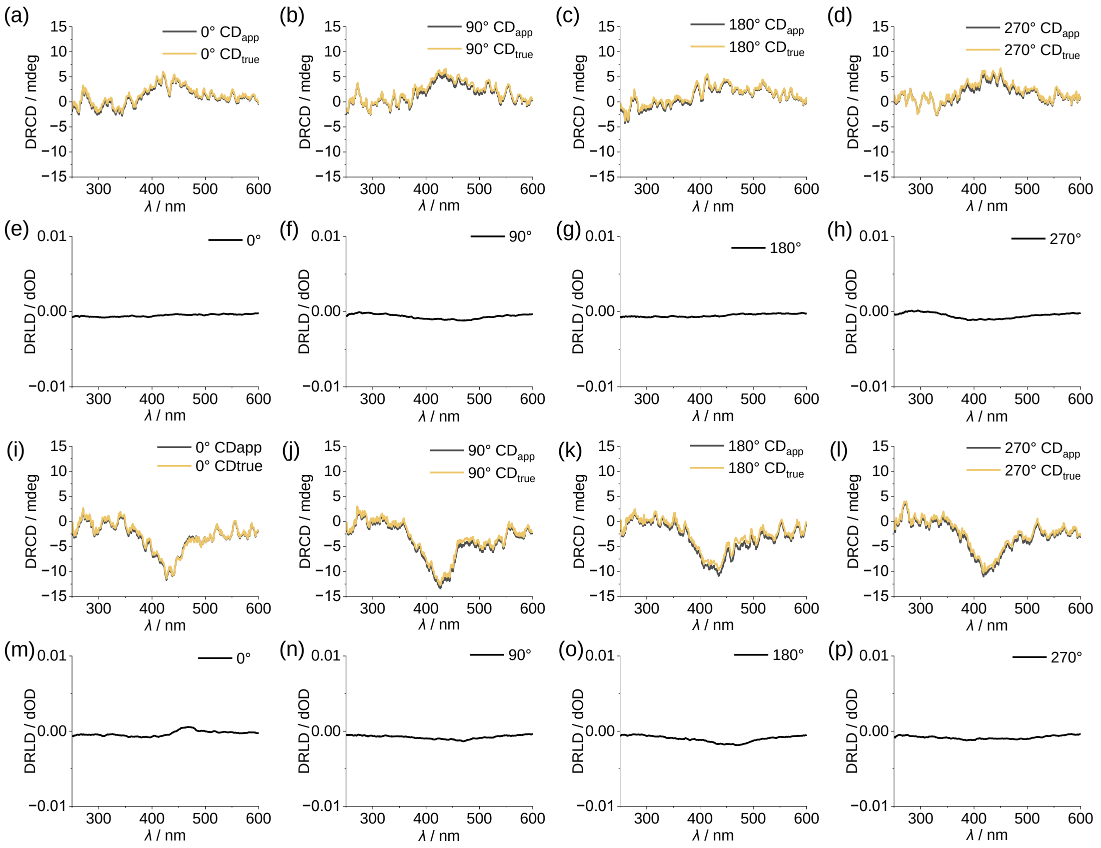

Figure S28. DRCD (upper) and relevant DRLD (below) spectra measured at different substrate angles from 0 ° to 270 ° of (a-h) ((+)-car)$_{1.9}$@(**1 2**) and (i-p) ((−)-car)$_{2.5}$@(**1 2**) microcrystals.

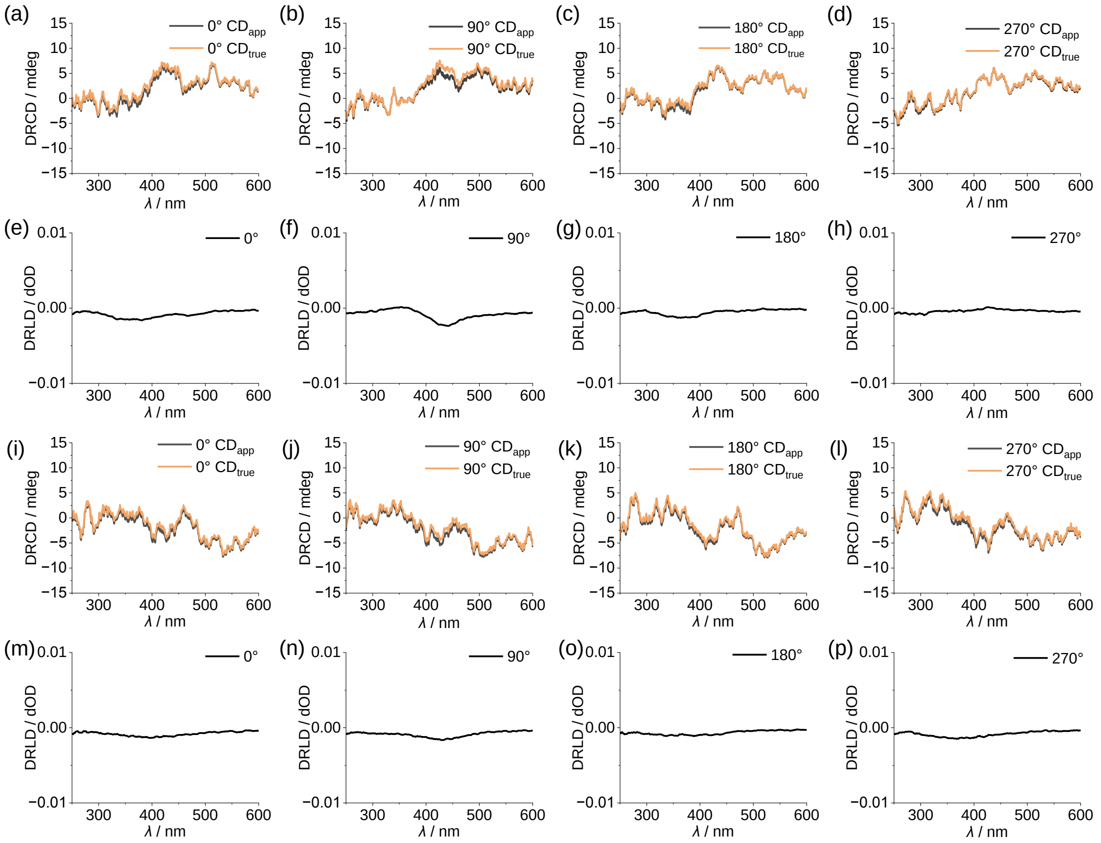

Figure S29. DRCD (upper) and relevant DRLD (below) spectra measured at different substrate angles from 0 ° to 270 ° of (a-h) ((+)-car)$_{1.3}$@(**1 2**) and (i-p) ((−)-car)$_{1.1}$@(**1 2**) microcrystals.

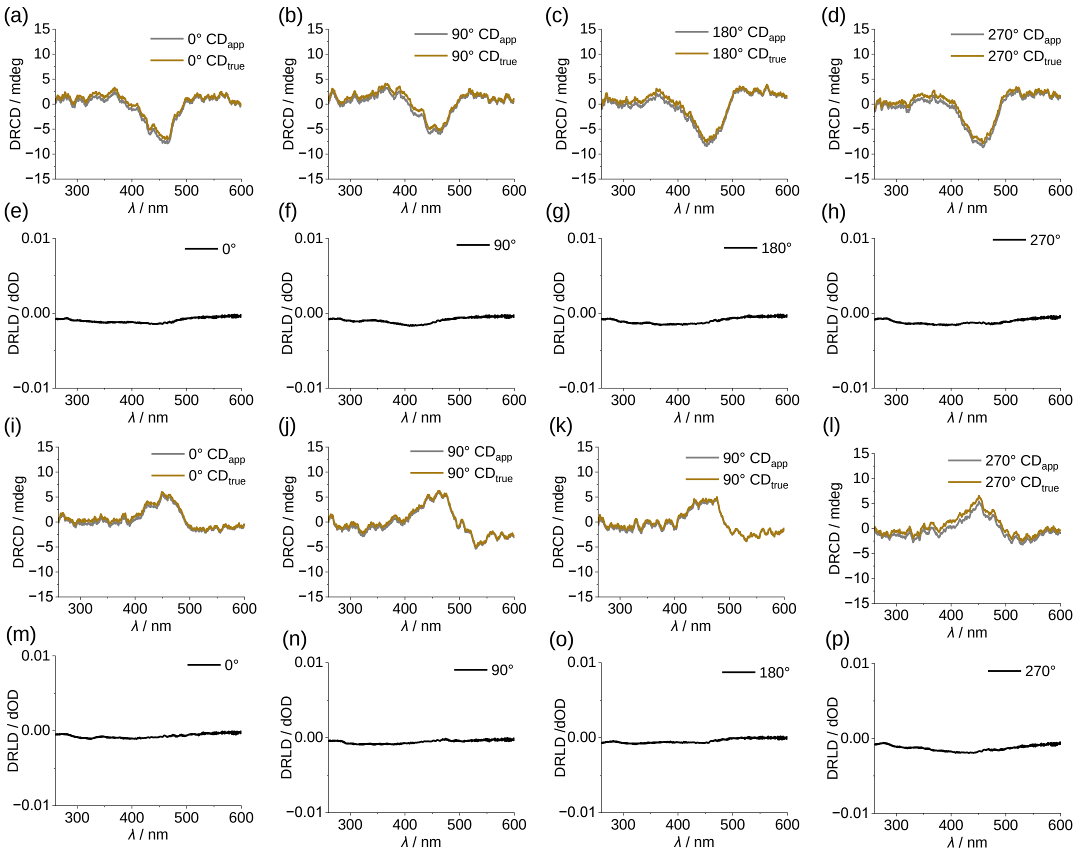

Figure S30. DRCD (upper) and relevant DRLD (below) spectra measured at different substrate angles from 0 °to 270 °of (a-h) ((+)-car)$_{<0.01}$@(**1**·**2**) and (i-p) ((−)-car)$_{<0.01}$@(**1**·**2**) microcrystals.

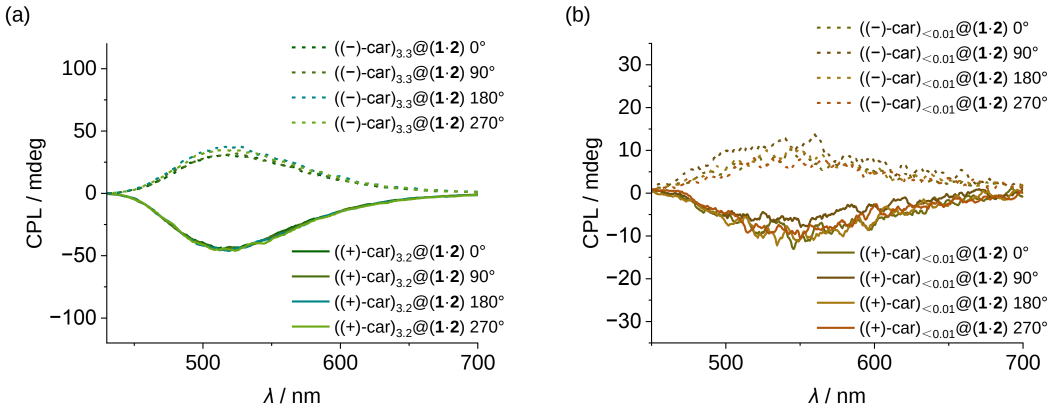

Figure S31. CPL spectra of (a) ((+)-car)$_{3.2}$@(**1**·**2**), ((−)-car)$_{3.3}$@(**1**·**2**) and (b) ((+)-car)$_{<0.01}$@(**1**·**2**), ((−)-car)$_{<0.01}$@(**1**·**2**) microcrystals measured at different substrate angles from 0 °to 270 °.

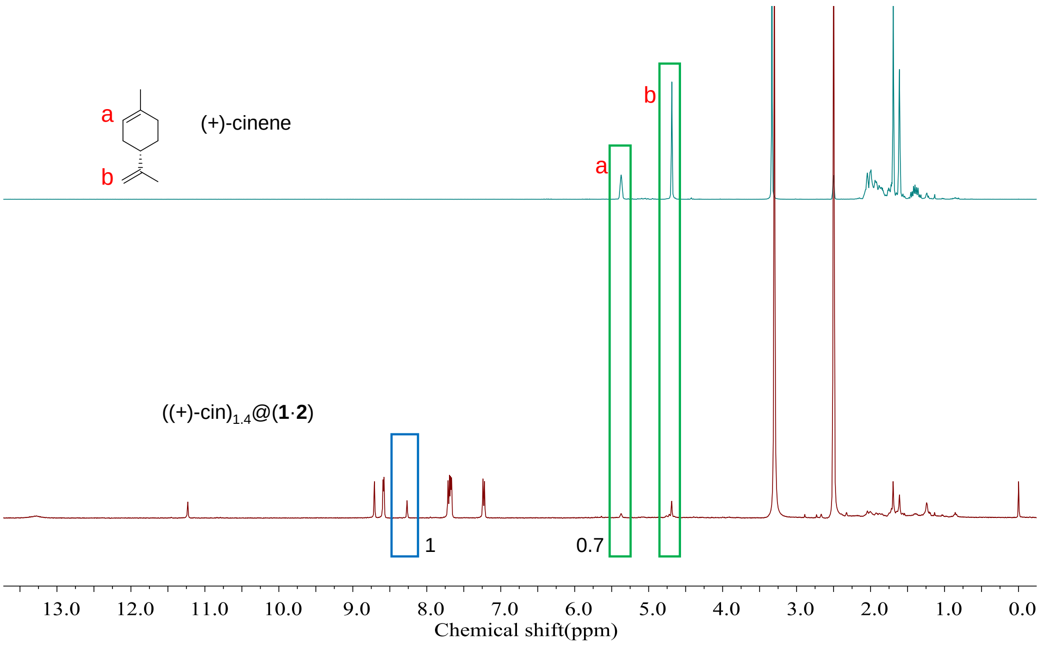

Figure S32. ^{1}H NMR spectra of (+)-cinene and ((+)-cin)$_{1.4}$@(**1 2**) in DMSO-d_6. The The navy-blue rectangle indicates one phenyl proton of the HOF framework. The green rectangles are associated with the proton signals from cinene.

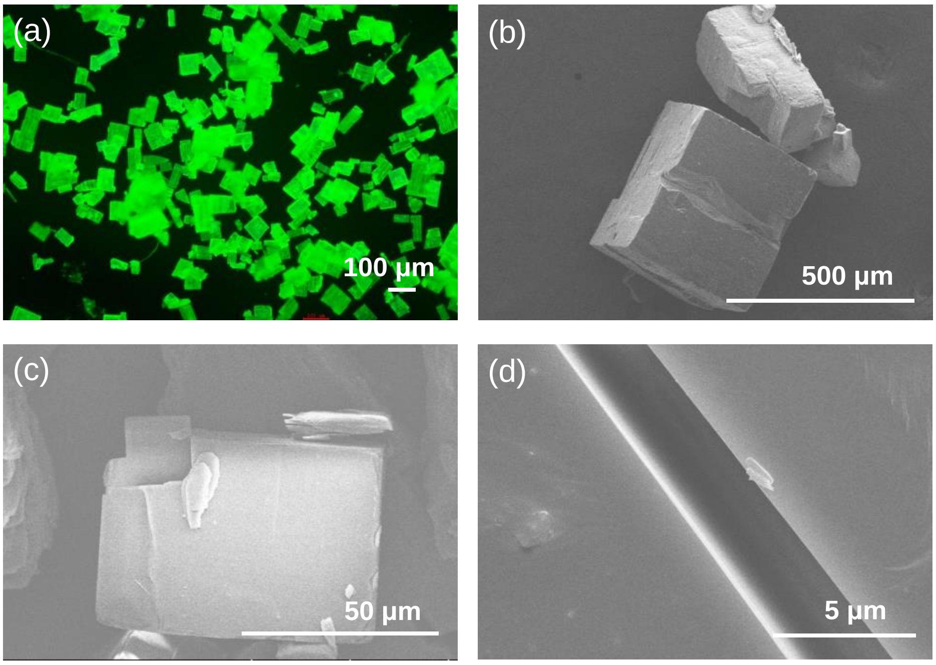

Figure S33. (a) Fluorescence microscopy and (b-d) SEM images of crystals of ((+)-cin)$_{1.4}$@(**1 2**).

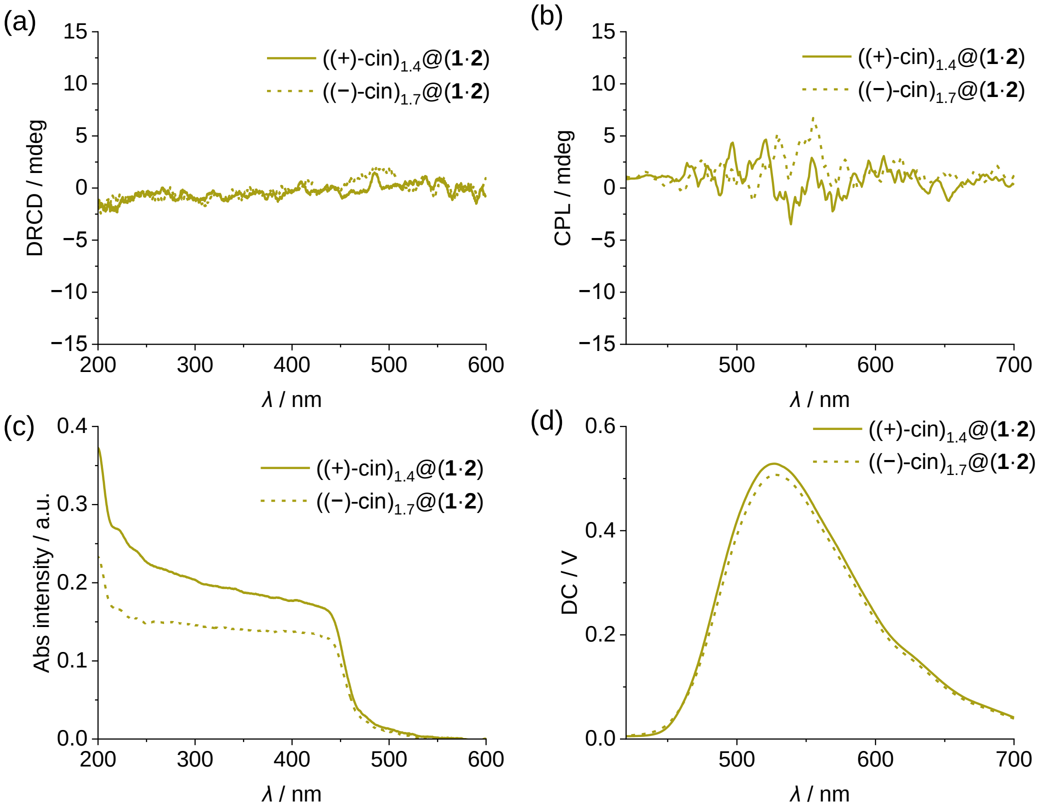

Figure S34. (a) DRCD, (b) CPL, (c) absorption and (d) emission spectra of the crystals of ((+)-cin)$_{1.4}$@(**1** **2**) and ((−)-cin)$_{1.7}$@(**1** **2**).

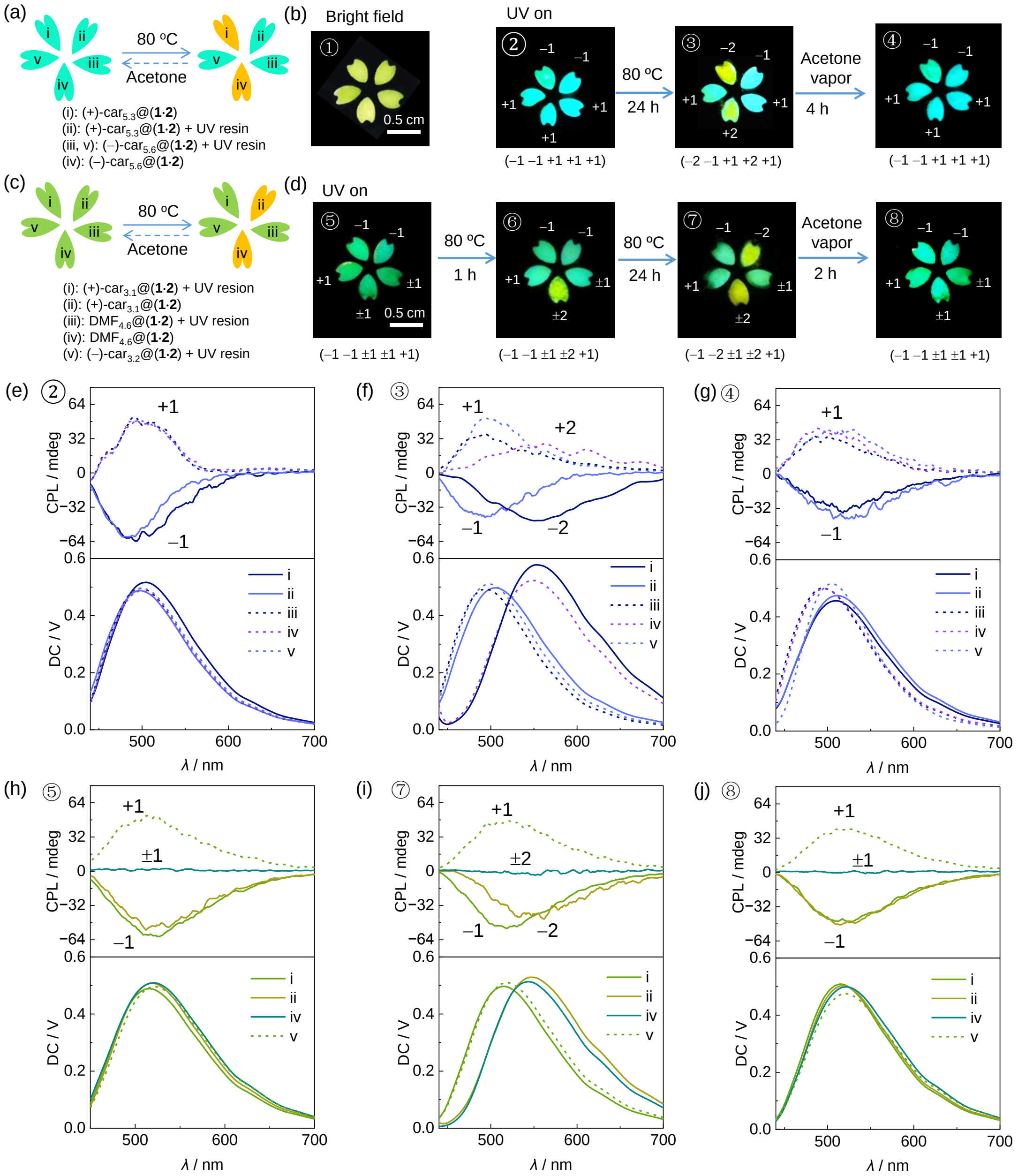

Figure S35. (a,c) Schematic representation of a reversible process by heating and acetone-fumigating a flower pattern with five petals (i – v) consisting of different compositions of microcrystals indicated. (b,d) Images taken during the implementation of the process shown in panel (a) and (c) respectively. Each image represents a five-number code shown in the parentheses, in which each number is sequentially determined by the optical state of petal (i – v). The number –1, –2, +1, +2, ±1, ±2 represents negative cyan/green CPL, negative yellow CPL, positive cyan/green CPL, positive yellow CPL, cyan/green emission with no CPL activity, and yellow emission with no CPL activity, respectively. (e – j) CPL (upper) and DC (lower) spectra of the different petals (i – v) of the image labeled with ②, ③, ④, ⑤, ⑦, and ⑧ respectively, displayed in panel (b, d).

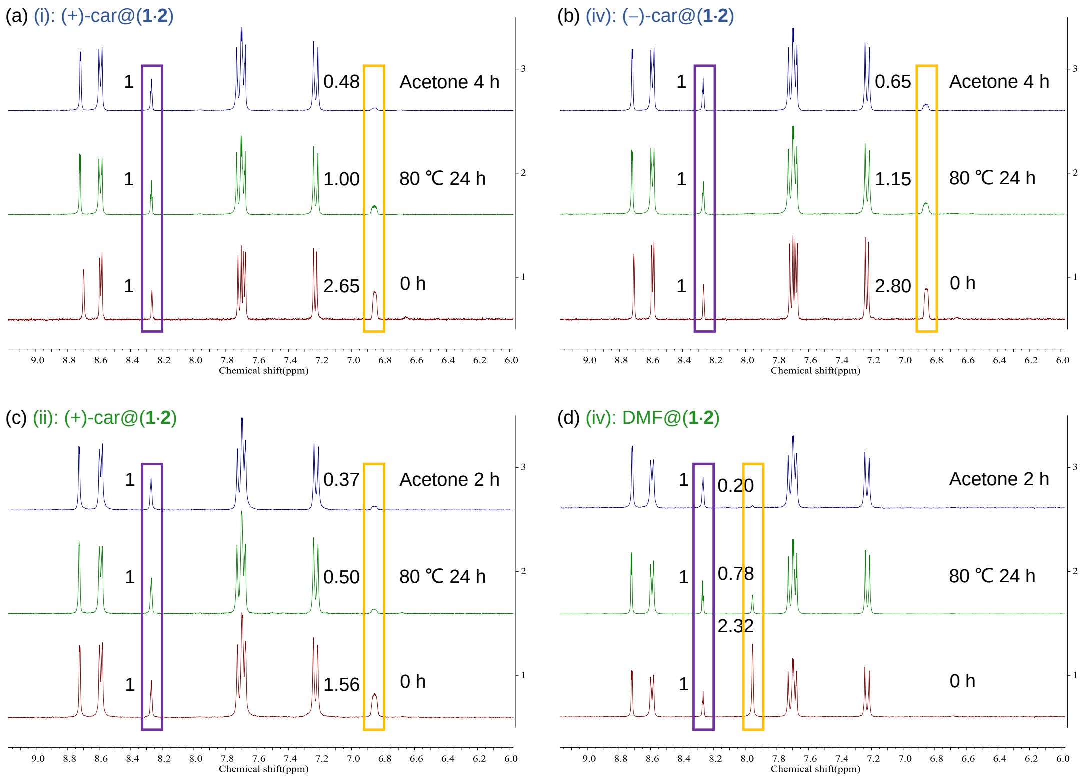

Figure S36. ^{1}H NMR spectral changes of (a) petal (i) and (b) petal (iv) shown in panel (a/b) of Figure S35 and (c) petal (ii) and (d) petal (iv) shown in panel (c/d) of Figure S35 during the reversible encryption process. The purple and yellow rectangle indicates one phenyl proton of the HOF framework and one olefin proton of carvone (a-c) or one amino proton of DMF (d), respectively.

References

(1) Mu, C.; Zhang, Z.; Hou, Y.; Liu, H.; Ma, L.; Li, X.; Ling, S.; He, G.; Zhang, M. Tetraphenylethylene-Based Multicomponent Emissive Metallacages as Solid-State Fluorescent Materials. *Angew. Chem., Int. Ed.* **2021**, *60* (22), 12293-12297.

(2) Lü, J.; Perez-Krap, C.; Suyetin, M.; Alsmail, N. H.; Yan, Y.; Yang, S.; Lewis, W.; Bichoutskaia, E.; Tang, C. C.; Blake, A. J.; et al. A Robust Binary Supramolecular Organic Framework (SOF) with High CO_2 Adsorption and Selectivity. *J. Am. Chem. Soc.* **2014**, *136* (37), 12828-12831.

(3) Li, Z.-Q.; Gong, Z.-L.; Shao, J.-Y.; Yao, J.; Zhong, Y.-W. Full-Color and White Circularly Polarized Luminescence of Hydrogen-Bonded Ionic Organic Microcrystals. *Angew. Chem., Int. Ed.* **2021**, *60* (26), 14595-14600.
